# Glandular trichome development, morphology, and maturation are influenced by plant age and genotype in high THC-containing cannabis (*Cannabis sativa* L.) inflorescences

**DOI:** 10.1186/s42238-023-00178-9

**Published:** 2023-04-04

**Authors:** Zamir K. Punja, Darren B. Sutton, Tommy Kim

**Affiliations:** 1 Department of Biological Sciences, Simon Fraser University, 8888 University Drive, Burnaby, BC V5A 1S6 Canada; 2 Department of Computing Sciences, Simon Fraser University, 8888 University Drive, Burnaby, BC V5A 1S6 Canada

**Keywords:** Cannabinoids, Cannabiaceae, Cannabidiol, Glandular trichomes, Marijuana, Resin production, Maturation, Scanning electron microscopy, Tetrahydrocannabinol

## Abstract

**Background:**

Glandular capitate trichomes which form on bract tissues of female inflorescences of high THC-containing *Cannabis sativa* L. plants are important sources of terpenes and cannabinoids. The influence of plant age and cannabis genotype on capitate trichome development, morphology, and maturation has not been extensively studied. Knowledge of the various developmental changes that occur in trichomes over time and the influence of genotype and plant age on distribution, numbers, and morphological features should lead to a better understanding of cannabis quality and consistency.

**Methods:**

Bract tissues of two genotypes—“Moby Dick” and “Space Queen”—were examined from 3 weeks to 8 weeks of flower development using light and scanning electron microscopy. Numbers of capitate trichomes on upper and lower bract surfaces were recorded at different positions within the inflorescence. Observations on distribution, extent of stalk formation, glandular head diameter, production of resin, and extent of dehiscence and senescence were made at various time points. The effects of post-harvesting handling and drying on trichome morphology were examined in an additional five genotypes.

**Results:**

Two glandular trichome types—bulbous and capitate (sessile or stalked)—were observed. Capitate trichome numbers and stalk length were significantly (*P* = 0.05) greater in “Space Queen” compared to “Moby Dick” at 3 and 6 weeks of flower development. Significantly more stalked-capitate trichomes were present on lower compared to upper bract surfaces at 6 weeks in both genotypes, while sessile-capitate trichomes predominated at 3 weeks. Epidermal and hypodermal cells elongated to different extents during stalk formation, producing significant variation in length (from 20 to 1100 μm). Glandular heads ranged from 40 to 110 μm in diameter. Maturation of stalked-capitate glandular heads was accompanied by a brown color development, reduced UV autofluorescence, and head senescence and dehiscence. Secreted resinous material from glandular heads appeared as droplets on the cuticular surface that caused many heads to stick together or collapse. Trichome morphology was affected by the drying process.

**Conclusion:**

Capitate trichome numbers, development, and degree of maturation were influenced by cannabis genotype and plant age. The observations of trichome development indicate that asynchronous formation leads to different stages of trichome maturity on bracts. Trichome stalk lengths also varied between the two genotypes selected for study as well as over time. The variability in developmental stage and maturation between genotypes can potentially lead to variation in total cannabinoid levels in final product. Post-harvest handling and drying were shown to affect trichome morphology.

**Supplementary Information:**

The online version contains supplementary material available at 10.1186/s42238-023-00178-9.

## Background

*Cannabis sativa* L., a member of the family Cannabaceae, is cultivated worldwide for production of fiber and seed (as hemp) and for its medicinal and psychotropic effects found in high THC-containing genotypes. In this study, “cannabis” is used to describe the high THC genotypes. Many cannabinoids, terpenes, and phenolic compounds are produced within glandular trichomes in *C. sativa* inflorescences (Elsohly and Slade 2005; Andre et al. 2016) and play important roles in the interaction of the plant with the abiotic and biotic environment (Wagener 1991; Schilmiller et al. 2008; Wang 2014). These phytochemicals, in particular delta9-tetrahydrocannabinol (THC) and cannabidiol (CBD), are synthesized from pre-cursor molecules of phenolics and terpenes by specific enzymes and accumulate within stalked glandular heads of trichomes. These trichomes are found primarily on bract tissues within female inflorescences (Fairbairn 1972; Kim and Mahlberg 1997; Mahlberg and Kim 2004), while leaves and stems bear sessile (nonstalked) glandular trichomes and nonglandular hairs (Turner et al.^ 1980^; Small 2017). The cannabis inflorescences are comprised of individual flowers clustered together in a raceme and are harvested after 7–8 weeks of development in the flowering phase of production. They are dried under specific temperature and relative humidity conditions for 4–5 days to achieve a moisture content of 10–12% prior to packaging and distribution to the medicinal and recreational markets.

There are many different genotypes of *C. sativa* (also referred to as chemotypes) (Welling et al. 2016; Lewis et al. 2017) that accumulate THC to different extents, ranging from 0.3% (hemp) to over 20% (expressed in relation to dry weight of the harvested female inflorescences or “buds”) in cannabis (Schwabe and McGlaughlin 2019; Zager et al. 2019). External factors, such as light levels and intensity, plant nutrition, and growing conditions, may influence the levels of THC in the inflorescences (Vanhove et al. 2011; Magagnini et al. 2018; Saloner and Bernstein 2021). Considering that trichomes are the sole sites for production of these commercially and medicinally important cannabinoids, knowledge of their development over time and the potential influence of genotype and plant age on distribution, numbers and developmental features is important to ensure consistent product quality. When glandular heads mature on trichomes, the color of the resin transitions from clear (translucent) to cloudy (milky white) and progresses to amber (brown) (Mahlberg and Kim 2004; Potter 2009). Morphological changes that may be taking place in trichomes during this maturation process have not been previously investigated. By understanding the factors affecting development and maturation of these trichomes, this information can potentially be used to monitor and assess quality aspects in this important medicinal plant both pre-and post-harvest.

The first objective of the present study was to determine the morphological changes taking place during development of glandular capitate trichomes, in two genotypes of cannabis, from 3 to 8 weeks of inflorescence development, using scanning electron microscopy. A second objective was to assess trichome numbers and distribution on bracts using light microscopy. For both objectives, we selected genotypes “Moby Dick” and “Space Queen” to provide a basis for comparison as they displayed differences in inflorescence morphology, density, and size, as well in as final THC accumulation in glandular heads, with “Moby Dick” yielding 10–12% and “Space Queen” in the range of 16–18% THC. Observations on trichome maturation and extrusion of resin and dehiscence of the heads are reported. A third objective was to investigate the effect of post-harvest handling practices and the impact of drying on glandular head integrity and numbers in five additional cannabis genotypes as an indication of product quality.

## Methods

### Plant growing conditions

Two high THC-containing genotypes of *C. sativa*—“Moby Dick” and “Space Queen”—were cultivated in a commercial indoor growing facility according to the regulations and guidelines issued by Health Canada under an MMPR—Marihuana for Medical Purposes—license prior to 2018. The plants were initiated from rooted cuttings and provided with the nutrient regime for hydroponic culture as described elsewhere (Punja and Rodriguez 2018). Lighting, temperature, and growth conditions were provided in accordance with industry production standards to ensure proper inflorescence development and maturity. The plants were first maintained in the vegetative stage for 2 weeks at a temperature range of 23–26 °C with an 18 h photoperiod and light intensity of 840 µmol.m^− 2^.s^− 1^. They were then transferred to an indoor flowering room under a modified photoperiod regime that consisted of a 12-h photoperiod with a light intensity of 1300 µmol.m^− 2^.s^− 1^ for an additional 8 weeks to the mature stage of flower development. The two genotypes were grown side-by-side under the same conditions throughout the duration of the experiment. The experiment was repeated over 3 consecutive cycles of plant production, each extending over a period of 12 weeks, in the same environment to provide replicate observations of the same genotypes. Inflorescences were removed for microscopic examination as described below.

### Sample collection for microscopic studies

**Fig. 1:**
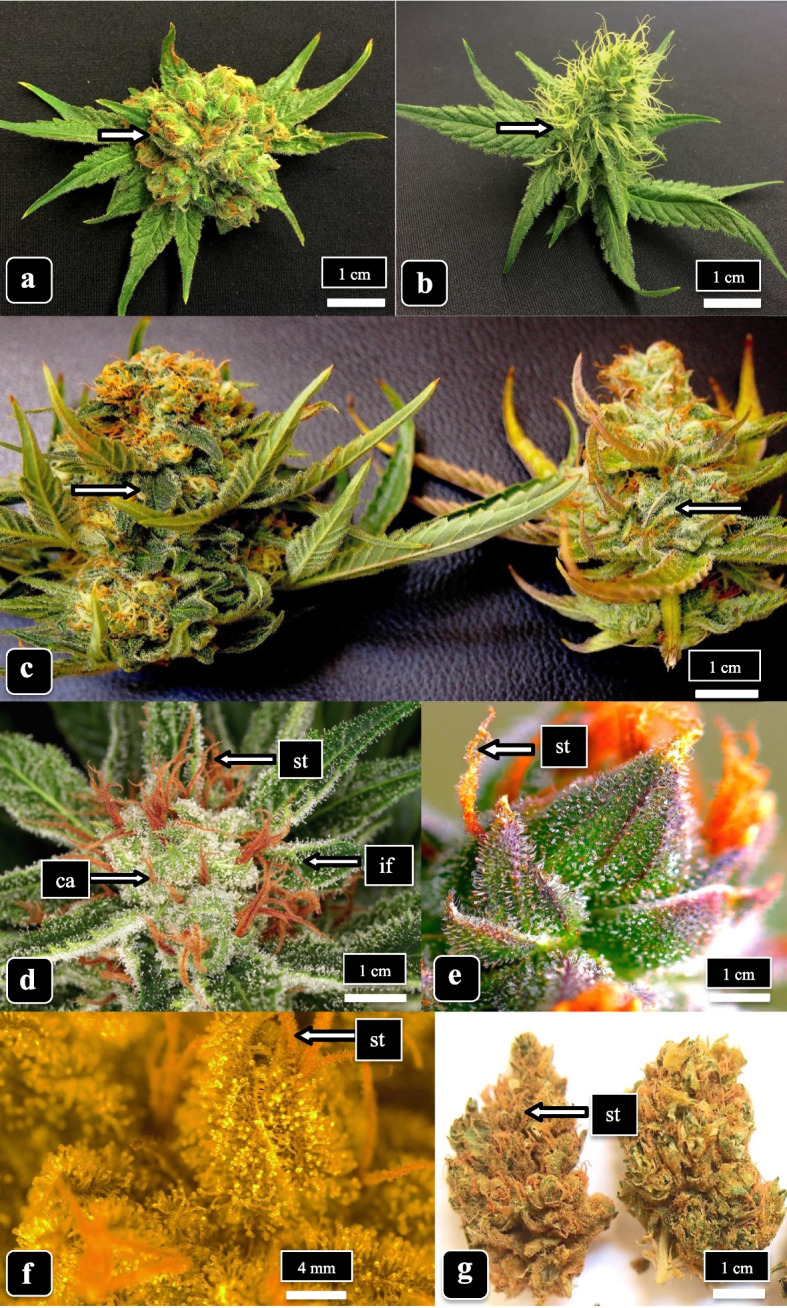
Morphology of developing inflorescences in the two cannabis genotypes examined in this study. **a **“Moby Dick” (MD) at 3 weeks of inflorescence development. **b **“Space Queen” (SQ) at 3 weeks. **c** MD (left) and SQ (right) at 6 weeks. The arrows in **a**–**c** show the positions at which bracts were sampled for microscopic observations. **d** Stigmas (st) appear orange-brown in color and trichomes can be seen on the calyx (ca.) and on inflorescence leaves (il) of strain MD at 8 weeks. **e**, **f** Trichomes on bracts of strain SQ at 8 weeks. The arrow points to stigmas (st) that appear orange-brown. **g** Dried inflorescence (bud) of MD after harvest. The bud has been trimmed to remove the inflorescence leaves. The arrow points to dried stigmas (st)

Single inflorescences (buds) were harvested from plants of genotypes “Moby Dick” and “Space Queen” at weekly intervals from 3 to 8 weeks of the flowering period. Buds were taken from a height of 1.7 m measured from the base of the plants, on two lateral branches bearing terminal inflorescences from each of three plants. At each sampling time, 2 buds were collected from each branch (*n* = 12) and gently placed in a plastic box lined with moist paper towels, sealed, and placed at 4 °C for no more than 48 h. For observation, bracts were gently cut with a sharp razor blade from three positions—top, middle, and bottom—from each inflorescence (Fig. 1a–d). The excised bracts, measuring 0.8–1 cm in length, were collected in duplicate at each position and prepared for scanning electron microscopy and light microscopy as described below (*n* = 24 for each genotype). According to Small (2017), bract tissues surrounding the calyx and those formed as small unifoliate leaves bear the highest density of trichomes.

### Trichome density measurements using light microscopy

Trichome density assessments were made on bracts collected as described above. Each bract was partitioned into interveinal segments demarcated by primary and secondary veins, as seen from each photographic image. The Polygon Lasso tool was used to trace the area of each segment using a 13 px line and the area was determined. They were examined at × 50 magnification under a SMZ-0171 stereomicroscope (Cole-Parmer, Ontario, Canada) and photographed with a Moticam 1080 digital Camera (Motic). All measurements were made using image analyzer Image J2 (Schneider et al. 2012). The position of each glandular trichome, representing the capitate form (stalked and sessile), was marked using a pen of 15 px thickness and then counted. This was repeated for three segments on each bract sample to provide replicate measures. The average trichome density on each bract was calculated as an average of the three segments. Trichome densities from the adaxial surface of bracts were used in these analyses. To compare differences between genotypes, position of bract, and effect of sampling time on trichome density, analysis of variance (ANOVA) was performed in Excel (Microsoft) and JMP (SAS) and means compared using Student’s *t-*test. A significance level of *P* = 0.05 was used.

### Scanning electron microscopy of trichomes

Small segments of bracts (0.5 × 0.5 mm^2^) from the inflorescences of both genotypes were prepared for scanning electron microscopy. Samples at 3 weeks and 6 weeks of the flowering period, representing early and mid-term flower development, and from 8 weeks, representing maturation, were examined. Both adaxial and abaxial surfaces were examined for each genotype. Tissue segments were adhered to a stub using a graphite-water colloidal mixture (G303 Colloidal Graphite, Agar Scientific, UK) and Tissue-Tek (O.C.T. Compound, Sakura Finetek, NL). The sample was submerged in a nitrogen slush for 10–20 s to rapidly freeze it. After freezing, the sample was placed in the preparation chamber of a Quorum PP3010T cryosystem attached to a FEI Helios NanoLab 650 scanning electron microscope (Thermo Fisher Scientific, Hillsboro, OR, USA). The frozen sample was sublimed for 5 min at − 80 °C, after which a thin layer of iridium (10 nm thickness) was sputter-coated onto the sample for 30 s at a current of 10 mA using a Leica EM ACE600 coater with iridium sputter and carbon evaporation functions (Leica Microsystems GmbH (Wetzlar, Germany). Cold fracturing was also used to obtain cross-sections through bract tissue to view development of trichome stalks, in which case a secondary coat of platinum was applied if required. The sample was moved into the SEM chamber and the electron beam was set to a current of 50 pA at 3 kV. Images were captured at a working distance of 4 mm, at a scanning resolution of 3072 × 2207 collected over 128 low-dose scanning passes with drift correction. Observations of trichome morphology and development were made from approximately 150 SEM images of each genotype. Measurements of trichome stalk length and glandular head size and the mean, variance and range were calculated from 25 images.

### Trichome color assessment

To capture the visual color change in the heads of capitate trichomes during maturation, ranging from clear to cloudy and transitioning to amber, bracts from inflorescences of genotype “Hash Plant” were photographed using a macroscopic lens and DSLR camera (Canon 5D mk. II, Canon, Japan). Samples from inflorescences at 3, 4, and 7 weeks of the flowering period were photographed. Five images were selected for each sampling time, inspected for clarity, and color calibrated. Images were resized to 2784 × 1856 pixels and converted from the RGB color space to the CIELAB color space for further analysis, similar to previous work where fruits were segmented from natural images (Murillo-Bracamontes et al. 2012). The CIELAB color space has separate channels for color and luminosity, unlike the RGB color space where color and luminosity information is combined. Capitate trichome heads were identified from each image based on detecting circular shapes in the CIELAB color channels using a handcrafted algorithm. The algorithm slides a second order Gaussian filter over the image and is tuned to detect circular objects of a specific pixel radius corresponding to trichome glandular heads (Supplementary Fig. 3). Once trichome glandular heads were identified from each image, they were automatically examined via the described color computations to assess trichome colors and circled.

Glandular head translucency was measured as the mean of the a-channel (red-green) coefficient of variation from all trichome glandular heads in each image. Trichome redness was measured by computing the mean red color component of all trichomes in each image. The red color component is the cosine of the color angle formed by ‘a’ and ‘b’ channels of the circular CIELAB color space and is robust to varying levels of color saturation (Supplementary Fig. 3). Data were averaged from 10 images from two replicates and means and standard deviations were graphed to show changes in translucency and red score over time. Data were compared for significant differences using ANOVA. For examination of trichome autoflorescence under UV light, samples were examined following the procedure of Livingston et al. (2020).

### Resin exudation from trichome heads and senescence

Scanning electron microscopic images of samples from inflorescences that were taken at 8 weeks of the flowering period were carefully examined under high magnification for evidence of droplets forming on the surface of glandular heads. These images were compared to those obtained from younger samples in which the droplets were absent. In addition, morphological changes in the appearance of the glandular heads exhibiting exudation were noted and included whether the cuticular surface was intact, whether the glandular head had shrunk or was shriveled, and if the entire head had collapsed.

### Trichome morphology on dried cannabis samples with hand and machine-trimming

To determine how post-harvest handling practices used commercially can affect trichome morphology and the proportion of trichomes with intact/detached glandular heads, samples of genotype “Pink Kush” were obtained from a drying room which provided forced air at temperatures of 21–24 °C and relative humidity of 50–55% for 5 days. The plants from which the inflorescence samples were obtained were grown under greenhouse conditions according to the methods described previously under the “Plant growing conditions” section. The samples differed in the method used to trim the inflorescence leaves – one sample was trimmed by hand, the other subjected to machine trimming (Punja et al. 2019). The samples were sputter-coated and observations were made using the SEM on the numbers of glandular heads that had broken off and those that remained intact. The experiment was conducted twice with a replicate batch of samples.

### Trichome morphology on dried cannabis samples of different genotypes

A comparison was made of inflorescences from four genotypes representing dried samples of “Skunk,” “Temple Spice,” “Healer,” and “Terpenade.” All samples were obtained from plants grown under the environmental conditions in the greenhouse as described under the “Plant growing conditions” section. All inflorescences were hand-trimmed and placed in a drying room which provided forced air at temperatures of 21–24 °C and relative humidity of 50–55% for 5 days and examined using the SEM after sputter-coating. Images were obtained at the same magnification and measurements were made from 25 trichomes in three replicate samples (*n* = 75). Observations made included trichome density (numbers per mm^2^), proportion of stalked to unstalked trichomes, and the proportion of trichomes that were dehisced (no glandular head) or senescent (glandular heads visibly shriveled). Observations were also made of the extent of resin secretion as evidenced by glandular heads sticking together or glands that had shriveled. The experiment was conducted twice with replicate samples.

## Results

### Morphology of inflorescences and trichome types

The inflorescences (flower buds) of cannabis genotypes “Moby Dick” (MD) and “Space Queen” (SQ) sampled at 3 and 6 weeks of the flowering period, and the approximate position from which bracts were sampled, are shown in Fig. 1a–d. As the buds matured, the color of stigmas changed from whitish-yellow (Fig. 1a, b) to reddish-brown (Fig. 1c, d). In 8 week old inflorescences, the bracts surrounding the calyx and the unifoliate inflorescence leaves contained a high density of trichomes when viewed under the dissecting microscope (Fig. 1e, f). After harvest and drying of the inflorescence of MD, the unifoliate inflorescence leaves curled inwards and the stigmas developed a deep tan-orange color (Fig. 1g).

**Fig. 2:**
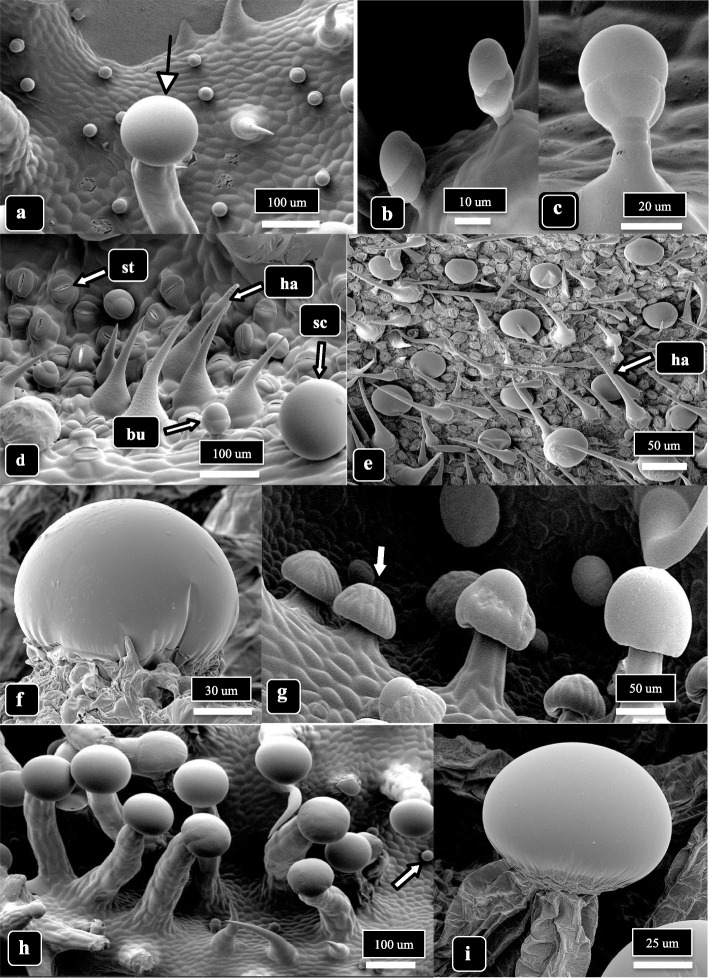
The different trichome types on *Cannabis* bract tissues observed in this study. All images are obtained from strain MD, lower bract surface, at 3 and 6 weeks of inflorescence development. **a** A stalked capitate trichome in the foreground (arrow) compared with small bulbous trichomes in the background. **b**, **c** Bulbous trichomes formed on short pedicels. **d** Nonglandular trichomes (ha) formed next to a bulbous (bu) and a sessile-capitate trichome (sc). In the background, abundant stomata (st) can be seen on raised epidermal cells. **e** Nonglandular hairs (ha) interspersed with sessile-capitate trichomes on a young bract. **f** Magnified view of a sessile-capitate trichome with fully formed head surrounded by a cuticle. **g** Presecretory sessile-capitate (arrow) and mature stalked-capitate trichomes with fully formed heads adjacent to one another. **h** Mature stalked-capitate trichomes formed in large numbers. Note the presence of small bulbous trichomes (arrow) and nonglandular trichomes in the background. **i** Magnified view of the head of a stalked-capitate trichome with fully formed cuticle surrounding the head

Under the cryo-scanning electron microscope, two glandular trichome types and nonglandular hairs were observed (Fig. 2). Small bulbous trichomes that were formed on short pedicels (10–15 μm in height) were present in both genotypes; the heads measured 15–30 μm in diameter (average of 25 measurements) (Fig. 1a–c). Nonglandular hairs were observed interspersed with sessile-capitate trichomes on 3-week-old bract tissues (Fig. 2d, e). The sessile-capitate trichomes, together with early development stalked-capitate trichomes, could be observed adjacent to one another (Fig. 2f, g). On 6-week-old bract tissues, fully developed stalked-capitate trichomes with enlarged heads surrounded by a cuticle were present (Fig. 2h, i).

### Comparison of genotypes

**Fig. 3:**
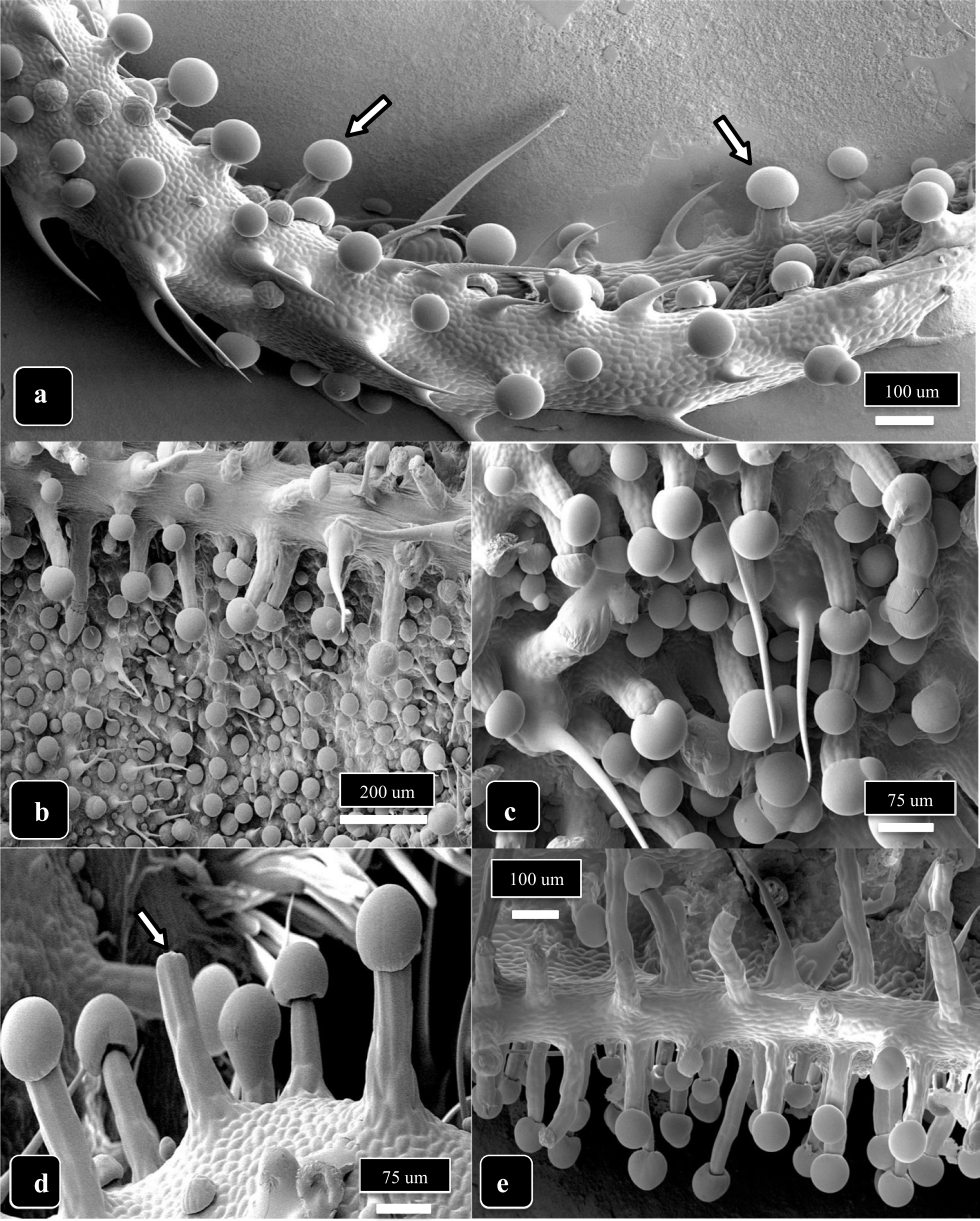
Capitate trichome development on cannabis strain MD, lower bract surface, at 3 and 6 weeks of inflorescence development. **a** At 3 weeks, most capitate trichomes at the tip of the bract are sessile but a few (arrows) have started to develop stalks. Nonglandular hairs can be seen. **b** Stalked-capitate trichomes forming first along the midvein of a bract; abundant sessile-capitate trichomes can be seen in the background together with nonglandular hairs. **c** At 6 weeks, stalked-capitate trichomes can be seen densely packed together. **d** A range of stalk lengths can be seen, with one trichome stalk on which the head has been detached (arrow). **e** A view of the edge of a bract at 6 weeks of inflorescence development, showing greater abundance of stalked-capitate trichomes on the lower compared to upper bract surface. Trichome stalk lengths are variable

Capitate trichome development on the lower bract surface of cannabis genotype MD, at 3 and 6 weeks of inflorescence development, is shown in Fig. 3. At 3 weeks, most capitate trichomes developing at the tip of the bract were sessile but a few had developed stalks (Fig. 3a). Stalked-capitate trichomes were first seen forming along the midvein of a bract, while the remainder of the tissues bore sessile-capitate trichomes (Fig. 3b). At 6 weeks, stalked-capitate trichomes were present in abundance and were densely packed together (Fig. 3c). A range of stalk lengths could be seen, and some heads had detached from the stalks (Fig. 3d). A greater number of stalked-capitate trichomes could be observed forming on the lower bract surface compared to upper bract surface (Fig. 3e). Trichome stalk lengths were variable.

**Fig. 4:**
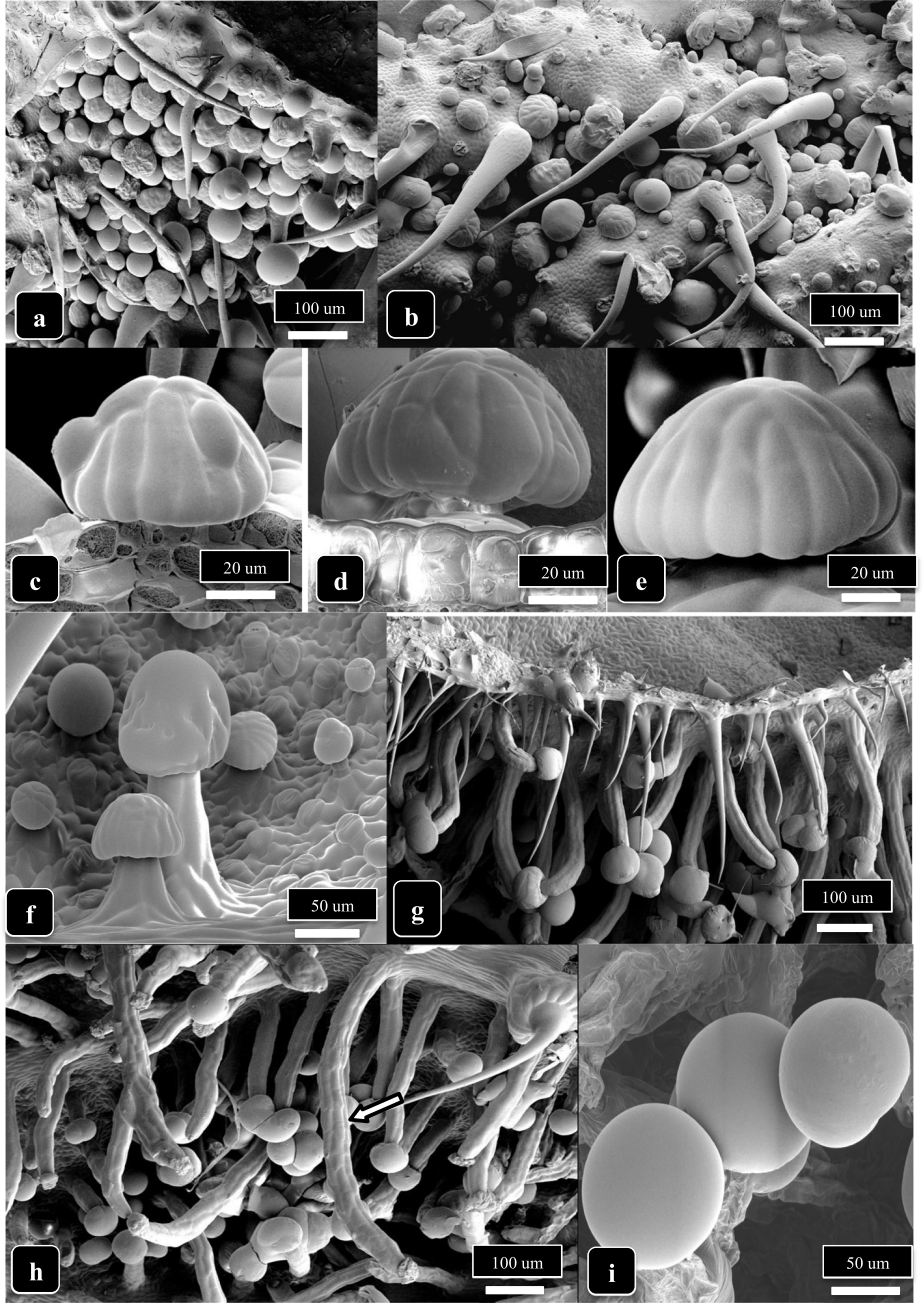
Capitate trichome development on cannabis strain SQ, lower bract surface, at 3 and 6 weeks of inflorescence development. **a** At 3 weeks, most capitate trichomes at the tip of the bract are sessile. **b** Close-up view showing a mixture of sessile-capitate trichomes with bulbous trichomes and nonglandular hairs. There are no stalked-capitate trichomes. **c**–**e** Presecretory (sessile) trichomes showing the outlines of presecretory disc cells. **f** A range of stalk lengths have developed on capitate trichomes, and sessile trichomes can be seen in the background. **g** At 6 weeks, stalked-capitate trichomes are densely packed together on the underside of the bracts and have formed long stalks. **h** Some stalked-capitate trichomes that form at the edge of a bract have extremely long stalks, up to 1000 μm (arrow). **i** Magnified view of capitate trichomes showing fully formed heads surrounded by a cuticle

Capitate trichome development on the lower bract surface of cannabis genotype SQ, at 3 and 6 weeks of inflorescence development, is shown in Fig. 4. At 3 weeks, most capitate trichomes on the bract were sessile-capitate and were present at a high density (Fig. 4a, b) There was a mixture of sessile-capitate trichomes with bulbous trichomes and nonglandular hairs on other bract tissues (Fig. 4b). A range of stalk lengths could be seen on capitate trichomes (Fig. 4f). At 6 weeks, stalked-capitate trichomes that formed along the edge of a bract had very long stalks, sometimes reaching up to 1,000 μm (Fig. 4g, h). The heads of capitate-stalked trichomes were fully formed at this time and surrounded by a cuticle (Fig. 4i). In Fig. 4c-e, presecretory (sessile) trichomes showing the outlines of presecretory disc cells could be seen. When young leaves of genotype SQ were examined under the scanning microscope, an abundance of nonglandular hairs was observed on the upper surface (Supplementary Fig. 1a, b). On older leaves, a mixture of nonglandular hairs together with sessile-capitate trichomes could be seen (Supplementary Fig. 1c, d). Bulbous trichomes were also present but there were no stalked-capitate trichomes observed.

### Trichome density and size measurements

**Fig. 5:**
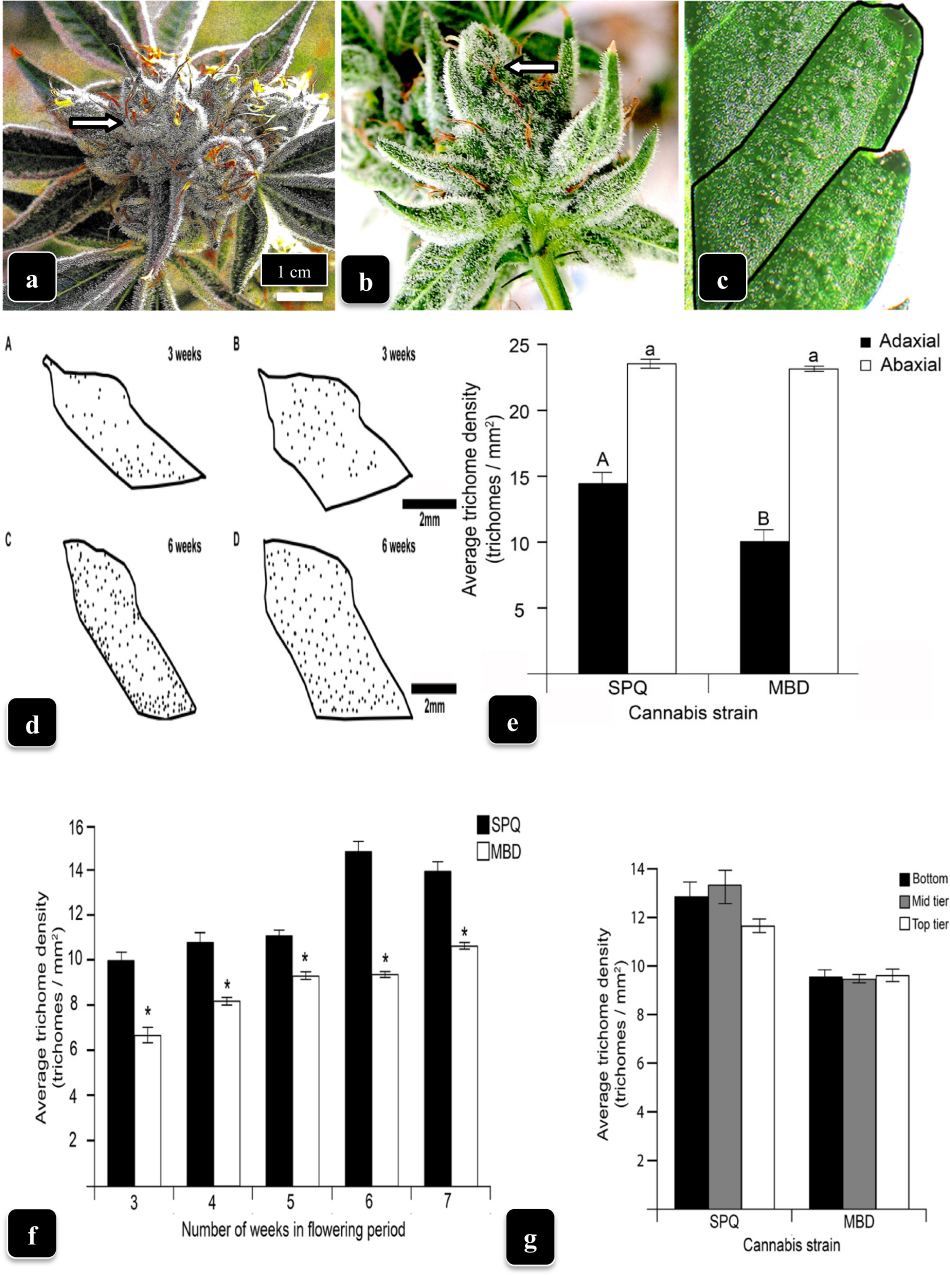
Bracts of cannabis genotype **a** SQ and **b** MD at 6 weeks of inflorescence development with abundant trichome development. Arrows point to approximate position where bracts were sampled. **c** To quantify trichome density, the bracts were excised, placed under the dissecting microscope (× 50 magnification) and the Polygon Lasso tool was used to trace the outline of each segment, as shown. The position of each capitate trichome was marked using a pen of 15 px thickness and then counted. **d** Spatial distribution of capitate trichomes on bracts of SQ (**A**, **C**) and MD (**B**, **D**) at 3 weeks and 6 weeks, respectively. Locations of trichomes are shown as black dots on bracts. **e** Density of capitate trichomes on the adaxial (upper) and abaxial (lower) surface of bracts at 6 weeks into the flowering period. Bars followed by different letters denote significant differences (*P* = 0.05, *t*-test). The lower bract surface contained more trichomes than the upper surface. **f** Average trichome density on upper bract surfaces as a function of number of weeks in the flowering period. Asterisk (*) denotes significant difference at *P* = 0.05 between the two genotypes according to ANOVA and Student’s *t*-test (*n* = 24). Genotype SQ produced significantly more trichomes than MD at all time periods. **g** Density of capitate trichomes on bracts as a function of position of sampling within the inflorescence (top, middle, bottom). Data are from week 6 of the flowering period. There were no significant differences due to sampling position. Vertical bars represent standard errors of the mean

To assess the pattern and density of trichome formation, bracts of genotypes MD and SQ (Fig. 5a, b) were excised and divided into segments demarcated by secondary veins (Fig. 5c) and traced out. The position of capitate trichomes viewed under the dissecting microscope was indicated by a dot (Fig. 5d). The spatial distribution of capitate trichomes on upper bract surfaces at 3 weeks and 6 weeks of development is shown in Fig. 5d. There was no obvious pattern of trichome distribution and they appeared to be randomly formed. The density of capitate trichomes on the adaxial (upper) and abaxial (lower) surface of bracts at 6 weeks into the flowering period is shown in Fig. 5e. The lower bract surface contained significantly (*P* = 0.05, *t*-test) more trichomes than the upper surface in both genotypes. The increase in glandular trichome density (numbers per mm^2^) on the upper bract surfaces of MD and SQ, from 3 weeks to 7 weeks, is shown in Fig. 5f. Trichome density increased gradually up to 6 weeks in both genotypes, and there was a slight decline in SQ in week 7. This was due to trichomes with senescent or detached glandular heads not being counted. Significantly more trichomes (*P* = 0.05, *t*-test) were produced at all sampling times in genotype SQ compared to MB. The average trichome density on upper bract surfaces was not significantly different for samples taken at the bottom, mid and top positions of inflorescences in both genotypes (Fig. 5f).

**Fig. 6:**
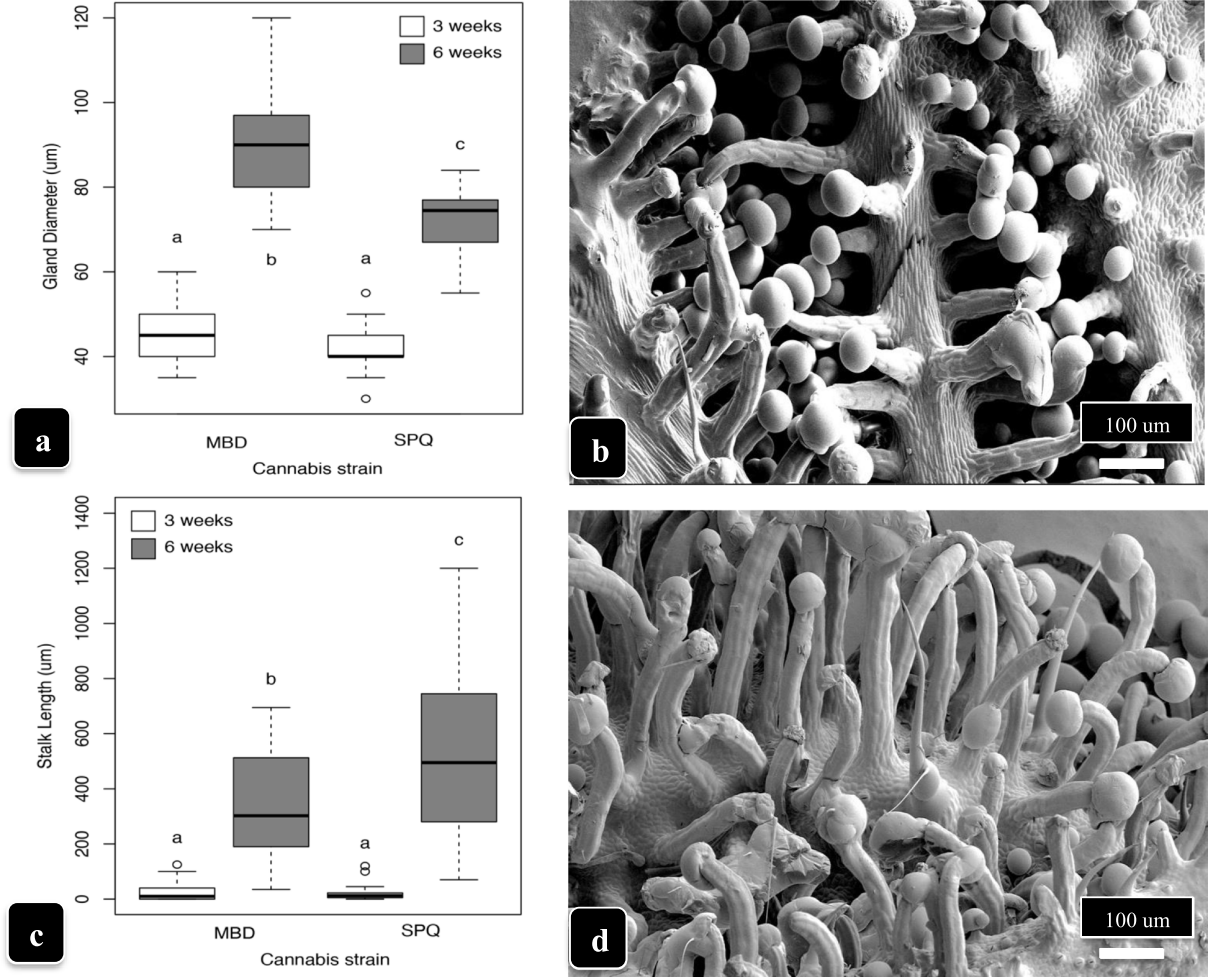
Comparison of stalked-capitate gland diameter (**a**) and stalk length (**c**) in genotypes MD and SQ at 3 weeks and 6 weeks of the flowering period. Bars followed by different letters indicate significant differences according to ANOVA followed post hoc by Tukey’s HSD test at *P* < 0.05 (*n* = 25). **a** Gland head diameter was significantly greater at 6 weeks compared to 3 weeks in both genotypes and in MD compared to SQ at 6 weeks. **c** Capitate trichome stalk length in genotypes MD and SQ at 3 weeks and 6 weeks of the flowering period. Bars followed by different letters indicate significant difference according to ANOVA followed post hoc by Tukey’s HSD test at *P* < 0.05 (*n* = 25). Stalk length was significantly greater at 6 weeks compared to 3 weeks and in SQ compared to MD at 6 weeks. **b**, **d** Development of stalked-capitate trichomes along the midveins of lower bract surfaces of cannabis genotype MD (**b**) compared to SQ (**d**) at 6 weeks of inflorescence development. Note the more extensive stalk development in SQ

Glandular head diameters and stalk lengths in genotypes MD and SQ at 3 and 6 weeks of development are shown in Fig. 6. Despite variability in the measurements due to asynchronous development of the trichomes, mean glandular head diameter was greater in MD compared to SQ at 6 weeks (90 μm vs. 75 μm, average of 25 measurements); there were no significant differences at 3 weeks (Fig. 6a). Stalk lengths were greater in SQ compared MD at 6 weeks (500 μm vs. 300 μm) but not at 3 weeks (Fig. 6c). Glandular head diameter increased in parallel with trichome stalk lengths as trichome development progressed from 3 to 6 weeks. The differences in appearance of the trichomes in the two genotypes at 6 weeks of inflorescence development are shown in Fig. 6 (b, d). The greater stalk lengths in SQ and the larger head size in MD are apparent.

### Stalk development on trichomes

**Fig. 7:**
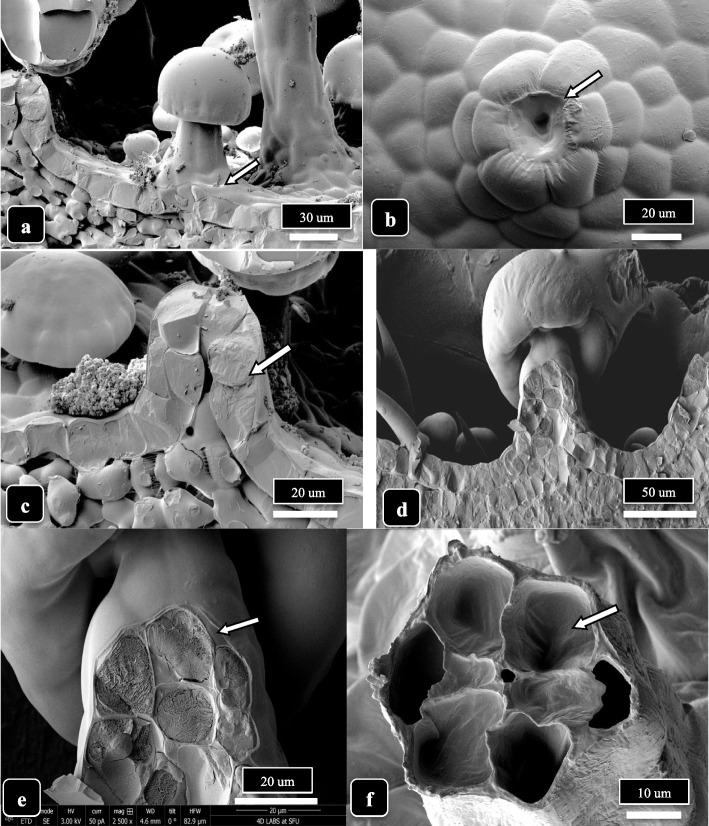
Trichome stalk elongation in capitate trichomes of cannabis genotype MD. **a** Early development of a stalk arising from extension of the epidermal cells of the bract tissue (arrow). **b** A ring of six raised epidermal cells demarks the early initiation of the stalk. **c** The elongation of the stalk occurs by expansion and upward growth (arrow) of the epidermal cell layer as seen in this longitudinal section through a stalk. **d**, **e** Longitudinal sectional view of a fully formed stalk on a capitate trichome showing the involvement of epidermal and hypodermal cells to form the stalk. **f** Cross-sectional view of a stalk showing the organization of the six cells. The interior of the cells can be seen (arrow)

**Fig. 8:**
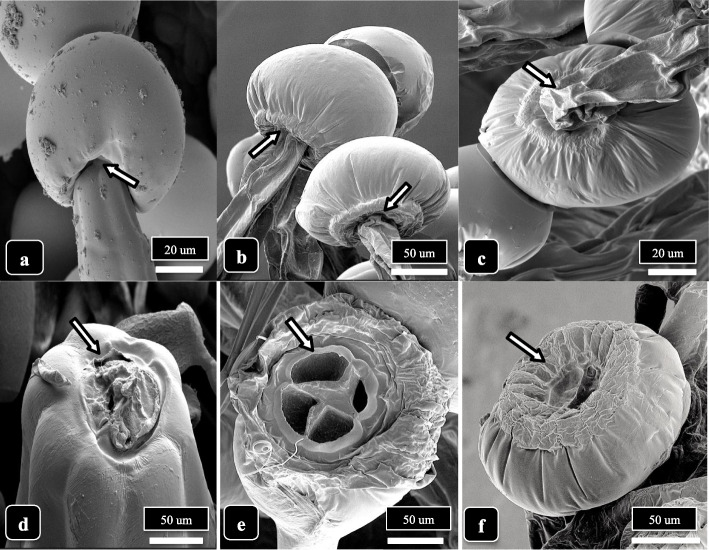
The attachment/detachment of capitate glandular heads to the trichome stalks are shown for genotype MD. **a** The stalk cells (arrow) progress into the head. **b**, **c** Attachment of the stalk to the glandular head (arrows) is shown in two dried samples. **d** A view of the top of the stalk showing the region (arrow) to which the glandular head would attach. **e**, **f** Views of the underside of detached glandular heads showing the appearance of the underlying region (arrows) that would be attached to the top of the trichome stalk

Stalks on capitate trichomes could be observed developing as extensions from the epidermal cell layer of bract tissues (Fig. 7a). The epidermal cells that comprised stalk growth can be seen forming a cluster of six cells that expanded vertically, leaving a central core (Fig. 7b). Both epidermal and hypodermal cells continued to divide and elongate to form a central column upon which the trichome gland was perched (Fig. 7c, d). The cuticular layer appeared to be continuous from the epidermal cell layer to the surface of the glandular head (Fig. 7d, e). A section through the fully developed stalk showed it to be comprised of six cells around a central core (Fig. 7e), and at the point of attachment to the underside of the glandular head, the tip of the stalk is constricted (Fig. 7f). The transition of the stalk into the base of the glandular head is shown in Fig. 8a. In dried cannabis samples, the cells of the stalk could be seen transitioning into the base of the glandular head (Fig. 8b, c). The top of the stalk revealed the site at which the glandular head was attached (Fig. 8d). A view of the undersurface of the glandular head showed a ring of stipe cells which would transition into the stalk (Fig. 8e) and the zone of separation appeared as concentric rings after the glandular head has detached (Fig. 8f).

### Resin exudation from trichomes and senescence

**Fig. 9:**
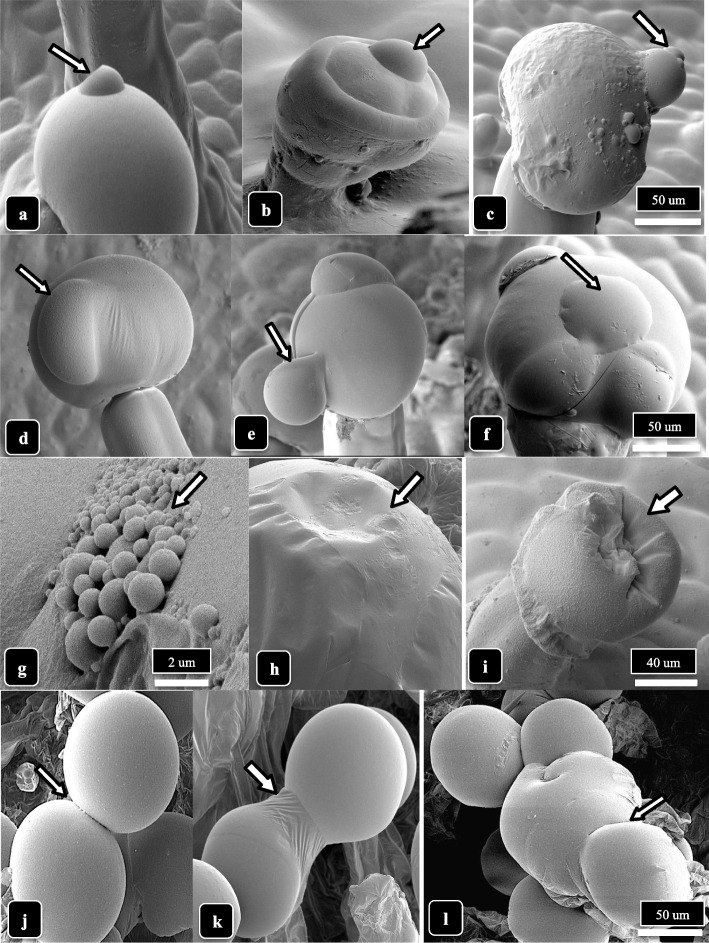
The process of resin extrusion and collapse of the capitate trichome heads is shown for cannabis genotype MD. Extrusion of resin and formation of droplets are shown by arrows. **a**, **b** Secretion from bulbous trichomes. **c**–**e** Secretion from capitate trichome heads. **f** Bulging of the cuticle (arrow) is due to resin secretion from the head. **g**–**i** Secretion of resin causes the cuticle to develop a central depression (arrows) and the trichome head to collapse. **j**–**l** Resin secretion causes trichome heads to stick to one another (arrows). Scale bars shown in **c**, **f**, **i**, **l** apply to the preceding images in each row

**Fig. 10:**
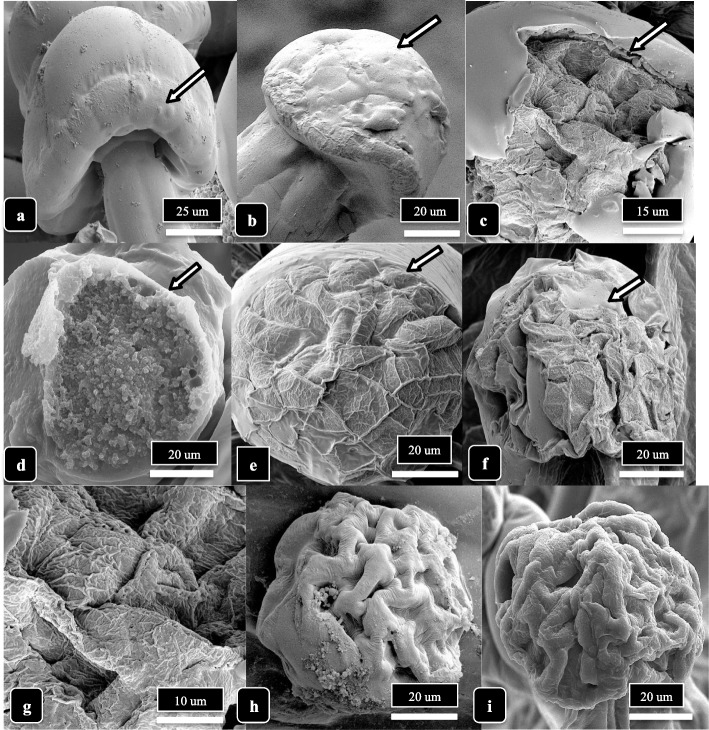
The process of trichome head senescence is shown. **a** Fully developed head with intact cuticle. No secretion of resin or shrinkage of the cuticle has occurred. Arrow points to the ring of secretory disc cells. **b** Shrinking of the cuticle and shriveling of the head has occurred, likely due to resin secretion. **c**, **d** Rupture of the cuticle (arrow) shows the dense granular appearance that is the resinous material underneath. **e**–**f** Shrinkage and collapse of the cuticle on mature glandular heads. The secretion of resin causes an aggregation of underlying resinous material and the surface of the head appears convoluted and wrinkled (arrows). **g** Close-up of dried resinous material. **h**, **i** Dried convoluted appearance of glandular heads that are completely senescent

Droplets presumed to contain resinous compounds were observed to be present on bulbous and capitate glandular heads on bract tissues of both genotypes at 6 weeks and 8 weeks of inflorescence development (Fig. 9). Small droplets were produced on bulbous trichomes (Fig. 9a, b) while larger droplets that created bulges on the cuticle surface were seen on stalked-capitate glandular heads (Fig. 9c, d). On some capitate glandular heads, several droplets had formed (Fig. 9e. f). In Fig. 9g and Supplementary Fig. 2, droplets exuded onto the cuticular surface of glandular heads could be clearly seen, each measuring approximately 2 μm in diameter. Following resin secretion, an inward collapse of the cuticle, presumed to have resulted from the release of contents contained within the head, was observed (Fig. 9h, i). The accumulation of resin on the surface of the head caused the cuticular surface to be sticky, resulting in the fusion of heads (Fig. 9j, k), sometimes producing aggregates containing 5 to 6 glandular heads (Fig. 9l). When compared to a glandular head in which resin secretion had not occurred and the cuticle was turgid (Fig. 10a), resin secretion caused the cuticle to wrinkle (Fig. 10b), and where the cuticle had been torn, the dense underlying contents of the head could be seen (Fig. 10c, d). With further secretions, the trichome head appeared shrunken (Fig. 10e, f), and eventually took on a wrinkled and dried appearance (Fig. 10h, i).

**Fig. 11:**
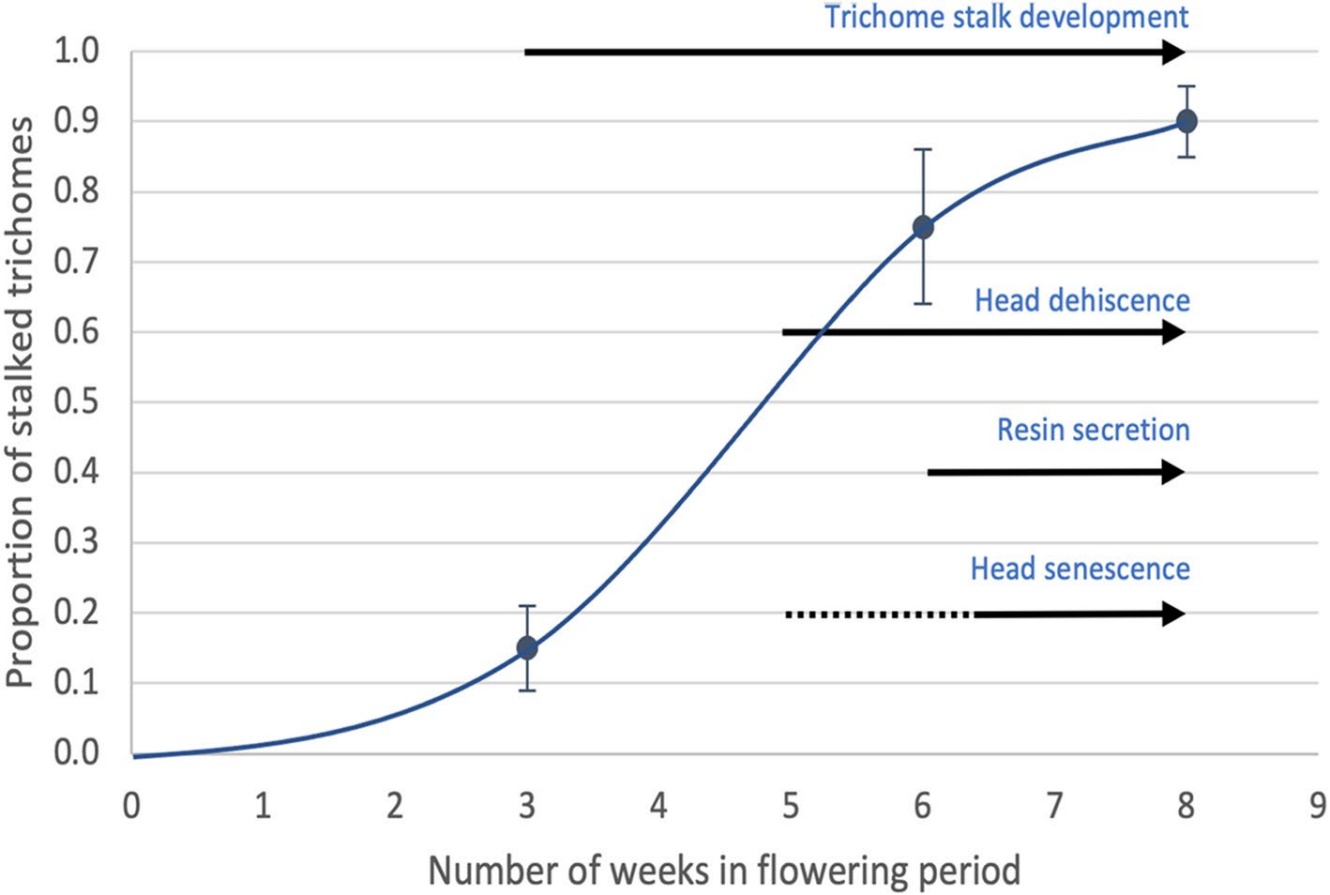
Schematic representation of the progression of capitate trichome development and senescence in cannabis genotypes MD and SQ based on the observations made in this study. The proportion of stalked-capitate trichomes increases exponentially with time in the flowering period to a maximum at 8 weeks. The process of glandular head dehiscence, secretion of resin and senescence begins at around 5 weeks and progresses to harvest (8 weeks). The timelines depicted here will vary by genotype as well as environmental conditions that impose stresses on the plant, which could accelerate the process of glandular head dehiscence and senescence

**Fig. 12:**
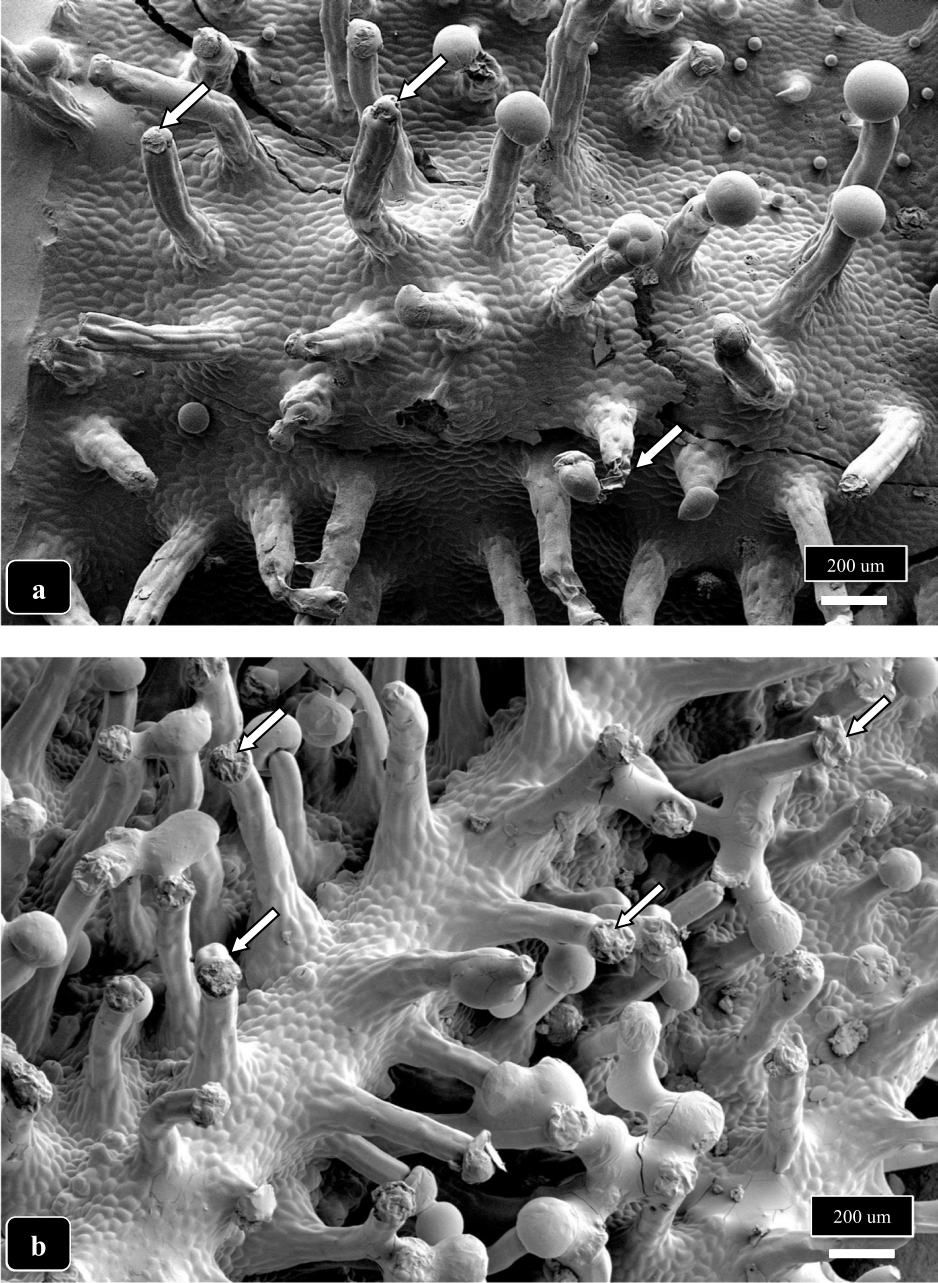
The occurrence of a large number of senescent capitate glandular heads on 8 week old bracts of cannabis genotypes MD (**a**) and SQ (**b**) is shown. In both samples, the glandular heads appear shriveled and shrunken, indicating a loss of resin has occurred (arrows)

A schematic representation of the progression of capitate trichome development and senescence in cannabis genotypes MD and SQ based on the observations made in this study is shown in Fig. 11. The proportion of stalked-capitate trichomes increases exponentially with time in the flowering period to a maximum at 8 weeks. The process of trichome head dehiscence, secretion of resin and dehiscence begins at around 5 weeks and progresses to harvest (8 weeks). The timelines depicted here will vary by genotype as well as environmental conditions that impose stresses on the plant, which could accelerate the process of glandular head dehiscence and senescence. A view of the senescing trichomes and the corresponding collapsed and dried heads on both genotypes MD and SQ in week 8 of the inflorescence developmental stage is shown in Fig. 12a, b. The aggregation of trichome heads as a result of resin secretion can also be seen (Fig. 12b).

### Trichome color assessment

**Fig. 13:**
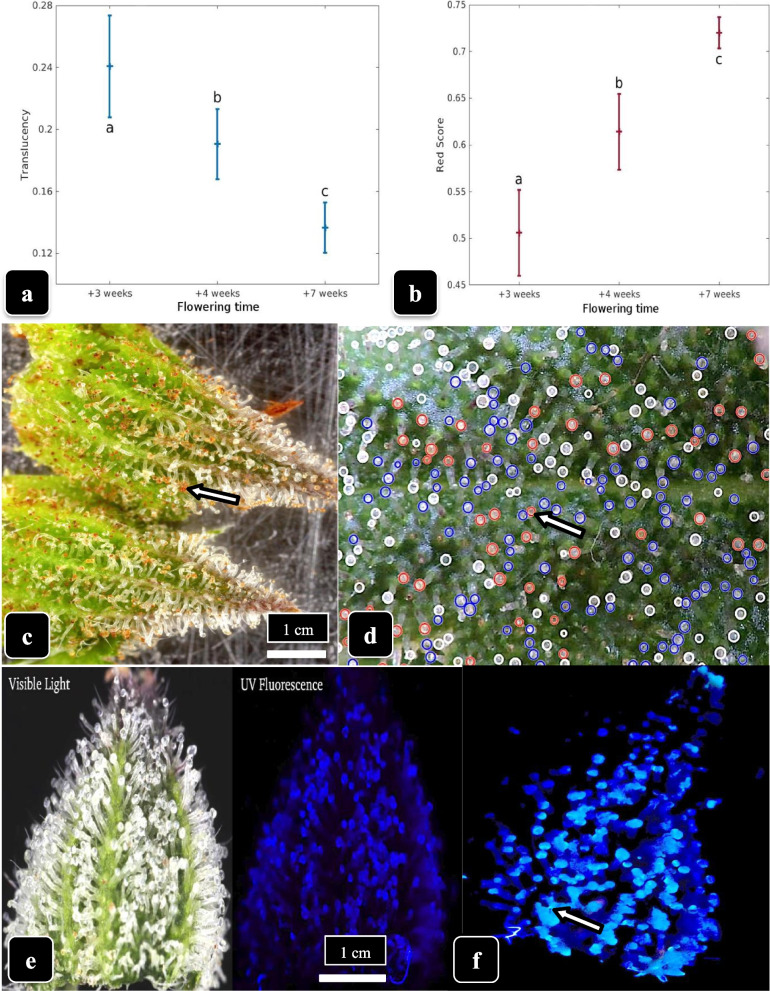
Assessing trichome maturation in cannabis. **a** Changes in translucency and **b** changes in red score in trichome heads of genotype “Hash Plant” progressing from 3 weeks to 7 weeks of the flowering period. A sharp reduction in translucency and a rise in red score can be seen. **c** Bract tissues in week 8 of the flowering period in which reddish-brown stalked-capitate glandular heads can be seen (arrow), especially on the lower half of the bract. **d** A representation of the proportion of glandular heads that are clear (blue circles), translucent (white circles) and red-brown (red circles) using the CIELAB color space. **e** Comparison of the appearance of stalked-capitate glandular heads under visible light (left) and under UV light (right) showing blue autofluorescence that is associated with the glandular head. **f** Close-up of glandular heads with an enhanced exposure showing autofluorescence under UV light. Some aggregation of the glandular heads due to resin secretion can be seen (arrow)

The extent of visible browning of the glandular heads was estimated during weeks 3 to 7 of the flowering period in genotype “Hash Plant.” The separation of translucent and red score measurements using the CIELAB color space showed a significant decrease in translucency values accompanied by a significant increase in red score values progressing from weeks 3, 5, and 7 of flowering time (Fig. 13a, b). A high proportion of glandular heads was visibly brown at 8 weeks (Fig. 13c). The CIELAB color space was used to identify clear, translucent, and brown glandular heads, resulting in white, blue, and red circles, respectively, being imposed on the image (Fig. 13d, Supplementary Fig. 3). In Fig. 13d, the number of glandular heads identified as clear, milky and brown were 83, 96 and 52, respectively. When bracts were examined under UV light, the glandular heads emitted a blue fluorescence (Fig. 13e), which under higher magnification could clearly be seen originating from the glandular heads (Fig. 13f).

### Trichome morphology on dried cannabis samples with hand and machine-trimming

**Fig. 14:**
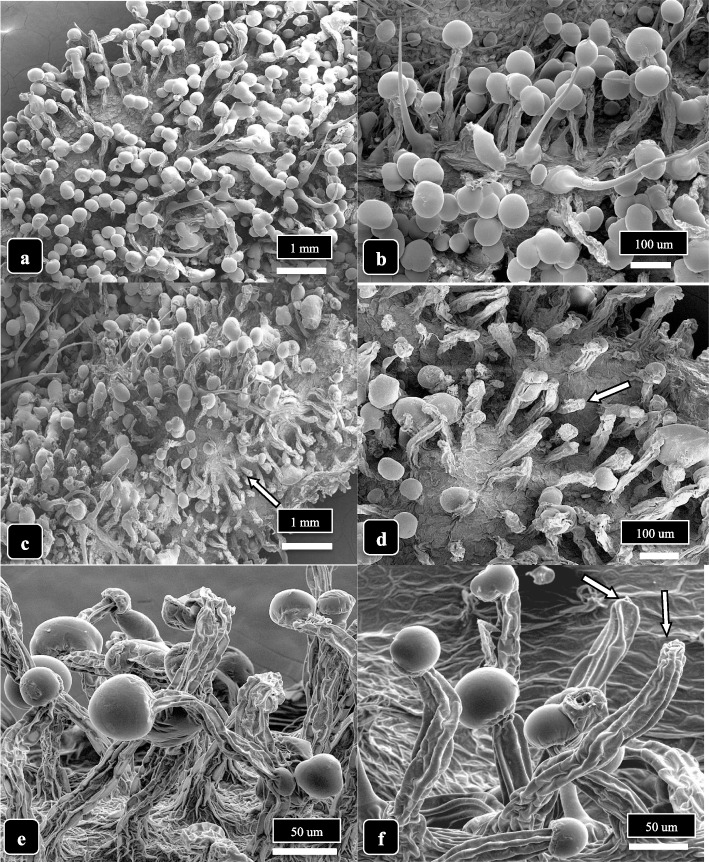
The effect of post-harvest handling practice on capitate trichome integrity of cannabis genotype “Pink Kush.” **a** A sample that was hand-trimmed to remove the inflorescence leaves before drying shows the majority of trichomes are intact and glandular heads are present. **b** Close-up view of trichomes in a hand-trimmed sample showing all glandular heads are intact. **c **A sample that was mechanically-trimmed to remove the inflorescence leaves shows a loss of trichomes and many heads have become detached (arrows). **d** Close-up view of trichomes in a mechanically trimmed sample showing many glandular heads are broken off or shriveled (arrow), especially at the edges of the bracts. **e**, **f** The dried stalks after 5 days of drying at 50–55% relative humidity show the outlines of the epidermal/hypodermal cells that make up the stalk. Most glandular heads are intact; a few have broken off (arrows). The cuticle is intact

In genotype “Pink Kush” samples in which the process of removal of inflorescence leaves was performed by hand-trimming (Fig. 14a, b) or by machine-trimming (Fig. 14c, d) followed by drying, were compared. Observations were made of the frequency of glandular heads that were intact on the stalks and any evidence of damage was recorded. In the sample in which the trimming process was done by hand, glandular heads were intact and there was little evidence of physical damage (Fig. 14a, b). In contrast, in samples subjected to machine-trimming, many glandular heads had become detached or were collapsed (Fig. 14c, d), leaving behind the bare stalks. In general, while the drying process caused trichome stalks to appear shriveled and twisted (Fig. 14e, f), the majority of the glandular heads were not shriveled and the cuticle was not damaged.

### Trichome morphology on dried cannabis samples of different genotypes

**Fig. 15:**
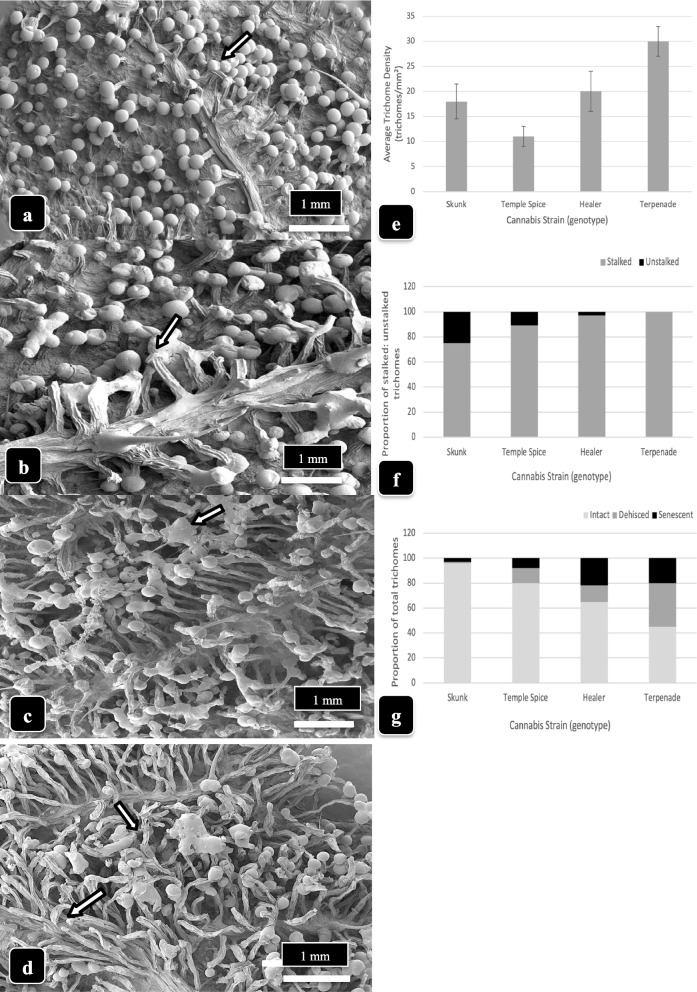
A comparison of capitate trichome density and maturation in four cannabis genotypes that have undergone the post-harvest drying process. All samples were hand-trimmed and images were obtained at the same magnification. Data were recorded from 25 trichomes in three replicate samples (*n*=75). **a **Genotype ‘Skunk’. Glandular heads are fully formed, trichome stalks are short, and no resin is released (arrow). **b **Genotype ‘Temple Spice’. Trichome stalks are intermediate in length and heads are releasing  resin (arrow). **c **Genotype ‘Healer’. Trichome stalks are long, most have secreted resin (arrow). **d **Genotype ‘Terpenade’. Trichome stalks are long, many glandular heads have released resin, and many heads have detached. **e **Comparison of trichome density in four cannabis genotypes based on data collected directly from SEM images. Standard errors of the means are shown. **f** Comparison of the proportion of stalked:unstalked trichomes in four genotypes. **g **Comparison of the proportion of dehisced and senescent trichomes in four genotypes

Inflorescences of four cannabis genotypes (“Skunk,” “Temple Spice,” “Healer,” and “Terpenade”) that had undergone commercial drying were examined by scanning electron microscopy (Fig. 15). Observations were made from representative and comparable bract tissues. In examining samples of genotype “Skunk” (Fig. 15a, e), a trichome density of about 18 per mm^2^ was obtained, with the proportion of stalked to sessile trichomes calculated at 0.79 (Fig. 15f). There were very few dehisced or senescent glandular heads in this genotype, and there was no evidence of resin secretion. In genotype “Temple Spice,” the average trichome density was 12 per mm^2^, and there was a low proportion (0.15) of sessile trichomes (Fig. 15b, f). A number of heads were stuck together, indicating resin secretion had occurred. In genotype “Healer,” the average trichome density was 20 per mm^2^, and the proportion of stalked trichomes was 0.99 (Fig. 15c, f). The proportion of dehisced:senescent trichomes was 0.38 (Fig. 15g). Finally, in genotype “Terpenade,” the trichome density was 30 per mm^2^ with no sessile trichomes visible and the proportion of dehisced to senescent heads was 0.68 (Fig. 15d, g). These observations indicate that trichome density and the proportion that were stalked and/or senescent at maturity varied with cannabis genotype.

## Discussion

Glandular trichomes in higher plants are morphologically represented by bulbous, peltate, and capitate types (Andre et al.^ 2016^). In *C. sativa*, the bulbous, sessile-capitate and stalked-capitate trichomes have been previously described (Hammond and Mahlberg 1977; Turner et al. 1980; Potter 2009; Andre et al. 2016; Raman et al. 2017; Livingston et al. 2020). In the present study, all three trichome types were present on bracts of both genotypes of *C. sativa* examined. The nonglandular trichomes (hairs) have been described previously in *C. sativa* (Fairbairn 1972; Dayanandan and Kaufman 1976)

The stalked-capitate trichomes have been very well-studied in cannabis because of their capacity to accumulate cannabinoid compounds and terpenes (Kim and Mahlberg 1997; Potter 2009; Andre et al. 2016; Livingston et al. 2020). These trichomes typically consist of one basal cell, one to many stalk cells, and a ring of secretory disc cells surmounted by a large sub-cuticular storage cavity (Ebersbach et al. 2018; Conneely et al. 2021). Several enzymes for cannabinoid and terpene biosynthesis, the most well-known of which are THCA synthase, CBDA synthase, and terpenes synthases, respectively, were shown to be present in capitate trichomes (Sirikantaramas et al. 2005; Andre et al. 2016; (Booth et al.^2017^)

In plants, trichome initiation is developmentally regulated (Turner et al. 1977; 1980; Maffei et al. 1986, 1989; Turner et al. 2000; Hauser 2014). Hammond and Mahlberg (1977) noted that bulbous and sessile-capitate glands in *C. sativa* arose early during bract growth while stalked-capitate glands arose somewhat later and first formed on the vein ridges and subsequently on interveinal regions of the bract. In peppermint, young leaves had fewer glandular trichomes than older leaves, while in other plant species, new glandular trichomes were produced continuously during leaf growth, resulting in glands of different ages occurring on the same leaf (Maffei 1986; 1989; Turner et al. 2000; Huchelmann et al. 2017). Our observations confirm that glandular trichomes are initiated throughout the period of cannabis inflorescence development. While this resulted in a general increase in numbers over time as the inflorescence matured, there were different developmental stages observed at any given time. The majority of trichomes at advanced stages of inflorescence development (6–8 weeks) were stalked-capitate (Fig. 11). There was also considerable variation in glandular head size and stage of development, and densities of stalked-capitate trichomes and stalk length were significantly different between the two genotypes examined. Turner et al. (1997; 1980) compared the densities of bulbous, sessile-capitate and stalked-capitate trichomes on the bracts of 3 cannabis strains. They reported that during early bract development, bulbose trichomes were present at a lower frequency than sessile-capitate trichomes. As bracts matured, the density of stalked-capitate trichomes increased over time to surpass the densities of the other two types. Our observations agree with these early findings (Hammond and Mahlberg 1997; Turner et al. 1980). On cannabis leaf tissues, bulbous, sessile-capitate trichomes and nonglandular hairs were observed (Supplementary Fig. 1), as noted in previous studies (Dayanandan and Kaufman (Dayanandan and Kaufman 1976)

The developmental regulation of glandular trichome formation and stalk development has not been previously studied in *Cannabis*. In general, the pattern and density of trichomes within natural populations of plants is regulated by a combination of endogenous developmental programs and external signals (Hauser 2014). In *Arabidopsis*, several genes and regulatory elements are associated with trichome development (Balkunde et al. 2010; Hauser 2014; Pattanaik et al. 2014; Matías-Hernández et al. 2016; Huchelmann et al. 2017; Tian et al. 2017; Hua et al. 2022). Specific mutations in these genes can alter the density of trichomes (Bloomer et al. 2012). In poplar plants, over-expression of the transcription factor MYB homologue increased trichome density (Plett et al. 2010). In addition, other transcription factors and micro-RNAs (miRNAs) may be involved in regulating the initiation, growth, and development of plant trichomes (Gan et al. 2017; Tian et al. 2017; Wang et al. 2021). In hop inflorescences (cones), peltate glandular trichomes (lupulin glands) produce flavonoids, bitter acids, and oils. A transcriptomic analysis of hop cones revealed an upregulated expression of trichome-development genes, which included transcription factors and homeodomain-leucine zipper proteins and phytohormone-related genes that are involved in trichome differentiation and development in other plant species (Mishra et al. 2020). The potential roles of these genetic factors in trichome development in cannabis have not been explored. There is likely to be an interplay of floral-regulating genes with hormones during trichome initiation in *Cannabis*, since capitate trichomes are primarily produced during maturation of floral bract tissues and their initiation is triggered by the onset of flowering.

In many plants, trichome initiation, development and density is enhanced by hormones such as gibberellic acid (GA), jasmonic acid (JA), and cytokinins (CK) (An et al. 2011; Glas et al. 2012; Hauser 2014; Tian et al. 2017; Huchelmann et al. 2017; Matías-Hernández et al. 2016; Wang et al. 2021; Hua et al. 2022). For example, mutant plants of *Arabidopsis* in which GA biosynthesis genes were knocked down, resulting in lower GA levels, developed fewer trichomes on leaves (Chien and Sussex 1996; Traw and Bergelson 2003). Alternatively, when GA was exogenously sprayed on an *Arabidopsis* wild-type plant, rosette leaf adaxial trichome production was significantly increased (Chien and Sussex 1996). Plants treated with GA biosynthesis inhibitors, such as paclobutrazol and uniconazole, did not produce trichomes (Chien and Sussex 1996; Perazza et al. 1998). Cytokinins are also able to stimulate trichome formation. Plants treated with benzylaminopurine (BAP) produced more trichomes on leaves, stems and flowers (Maes et al. 2008; Gan et al. 2017). In tomato, stress-induced jasmonic acid signaling induced trichome formation on sepals and leaves through interactions with zinc finger transcription factors (Hua et al. 2022). The interplay of these various hormones with trichome development and their potential to increase trichome densities and enhance cannabinoid levels in *Cannabis* have not yet been explored.

Environmental stresses, such as high salt, low temperatures, drought stress, heavy metal exposure, extremes of photoperiod, and exposure to UV-B light, can also affect trichome density in a range of plant species (Hauser 2014; Tian et al. 2017; Wang et al. 2021). On silver birch leaves, higher temperatures and water stress reduced the number of glandular trichomes on adaxial leaf surfaces but not on abaxial surfaces (Thitz et al. 2017). In white birch trees, defoliation in the preceding summer caused a shift from glandular to nonglandular trichome production the following year, suggesting a trade-off due to imposed stressful environmental conditions (Rautio et. 2002). Glandular trichomes in a number of plant species have been shown to play a role in reducing ozone toxicity and reduce ozone-induced stress (Li et al. 2018). The impact of environmental stresses on trichome production and potentially THC levels in *Cannabis* have not been explored.

It is known that the competency to enter the trichome pathway is limited to specific epidermal cells. Werker (2000) indicated that trichome development commences at an early stage of leaf development, often prior to development of stomata and sometimes even before the leaf primordia were distinguished. One epidermal cell gave rise to the trichome, originating from a protodermal cell that enlarges and divides. Subsequently, neighboring epidermal cells produce the stalk cells, which often formed in a circle, as observed in the present study. Once an epidermal precursor is specified to acquire trichome cell fate, a mechanism of lateral inhibition of the surrounding epidermal cells is initiated in *Arabidopsis* (Langdale 1998; Kirik et al. 2004). This lateral inhibition mechanism involves cell-to-cell communication, such that “trichome activation factors” can turn on negative regulators of trichome initiation, which subsequently move into neighboring epidermal pavement cells to prevent trichome formation. In addition, these trichome regulators are also able to move between cells. This elaborate and well-regulated genetic network forms a “trichome activator complex,” which interacts with GA and CK as described above, and can determine which epidermal cell may, or may not, morphogenetically develop into a trichome in *Arabidopsis* (Matías-Hernández et al. 2016; Feng et al. 2021). While *Cannabis* bracts seemingly appear to form trichomes randomly and asynchronously, further studies may reveal the cell-to-cell interactions that take place in these tissues to cause only specific epidermal cells to develop into trichome cells. It is unclear why trichomes appear earlier along mid-veins and edges of bracts in *Cannabis* or how differential timing of initiation may lead to asynchronous trichome development.

The developmental regulation of epidermal cell expansion to initiate trichome stalk formation and length are also not well understood (Huchelmann et al. 2017). In *Cannabis*, sessile-capitate trichomes on bracts, in which the stalk cells have not expanded, are considered to be a younger developmental stage of stalked-capitate trichomes (Hammond and Mahlberg 1977; Mahlberg and Kim 2004; Livingston et al. 2020). However, not all sessile-capitate trichomes progress to become stalked-capitate trichomes; therefore, bract tissues generally contain varying proportions of both. Glandular heads tend to enlarge before cell division in stalks is completed (Werker 2000); in *Cannabis*, this seems to occur between 3 weeks and 6 weeks of the flowering period. Enlargement of stalk cells is completed when the head cells have almost reached their full size (Werker 2000). This suggests that developmental regulation of stalk elongation is correlated with head maturity. Both glandular head diameter and stalk length were observed to increase from 3 weeks to 6 weeks of the flowering period in both genotypes examined in this study. However, stalk lengths were variable, with some reaching 1000 μm while other sessile-capitate trichomes never developed stalks. In tobacco, a proteomics study of short-stalked and tall glandular trichomes revealed that there were differential metabolic activities in both (Sallets et al. 2014). The genetic or environmental factors influencing trichome stalk length and the significance of extremely long trichome stalks on *Cannabis* bract tissues, as observed in genotype SQ in this study, remains unexplored. Since stalk cells originate from the epidermal cell layer, they contain chloroplasts and are photosynthetically active (Conneely et al. 2021). Proteomic analyses of glandular head and stalk cells in *Cannabis* revealed that many proteins in the former were involved in secondary metabolism and were distinct from those found in the latter, which were involved in transport processes and cellular respiration (Conneely et al. 2021). The glandular heads reportedly acted as “sinks” for carbon (sugar) utilization derived from photosynthesis, while the stalk cells and mesophyll cells in the bract tissues served as “sources” of carbon as they both contain chloroplasts (Conneely et al. 2021). Presumably, longer stalks would have a greater capacity to provide photosynthate to developing glandular heads compared to short-stalked trichomes, since glandular trichome stalks were reported to contribute to energy production, photosynthesis, and detoxification of metabolites (Conneely et al. 2021). Significantly higher levels of THC (by up to 20-fold) were reported to be present in stalked-capitate heads of cannabis compared to sessile-capitate heads, suggesting that cannabinoid accumulation also increased with stalk elongation and glandular head development (Mahlberg and Kim 2004). Livingston et al. (2020) described a strong correlation between trichome stalk elongation and increased numbers of secretory disc cells in glandular heads, which resulted in increased cannabinoid content as well as monoterpene and sesquiterpene accumulation. Therefore, inflorescences with a higher ratio of stalked:unstalked capitate trichomes would be expected to have a higher overall cannabinoid content. The interplay of glandular head development, stalk elongation, and THC accumulation in *Cannabis* deserves further investigation since it could have important commercial implications. For example, manipulations to promote a higher frequency of stalked-capitate trichomes or earlier elongation of stalks may result in higher THC levels.

Since cannabinoids accumulate primarily within the stalked-capitate glandular heads (Kim and Mahlberg 1997; Andre et al. 2016; Livingston et al. 2020), breeding efforts which have targeted increased total THC levels (Cascini et al. 2012) would likely be accompanied by increased trichome density and larger heads. Small and Naraine (2015) reported that glandular heads were larger (average diameter of 129 μm) in drug-type high-THC cannabis strains compared to low-THC hemp strains (80 μm), indicating selection through breeding efforts had resulted in increased volume of resin-containing glands. De Meijer et al. (2009) reported that mutant lines of *C. sativa* that formed very small glandular heads produced no cannabinoids. In hop inflorescences, the number and size of glandular trichomes was positively correlated with the concentrations of bitter acids and polyphenols (Patzak et al. 2015). However, in *Cannabis*, the relationship between increased trichome density and total cannabinoid levels has not been firmly established. By comparing additional genotypes with different trichome densities, or mutant lines that produce significantly lower trichome numbers, the relationship of trichome density to cannabinoid levels can be ascertained. In basil plants, Werker et al. (1993) correlated the gland density with essential oil content, while in hop cones, the number of glandular trichomes was positively correlated with content of alpha-acids (Srečec et al. 2011b). In menthol mint, increased light intensity positively affected trichome density (both peltate and bulbous trichomes) as well as essential oils (de Souza et al. 2016; 2020). The effects of environmental factors, such as temperature, nutritive status of the plants, and light quality and intensity on trichome density have not been established for *Cannabis*. A recent study (Saloner and Bernstein 2021) has shown that nitrogen fertilization impacts final THC levels in *Cannabis*; the effect on trichome density was not studied. The final levels of THC in *Cannabis* inflorescences are ultimately influenced by the strain or genotype (Vanhove et al. 2011). Similarly, hop genotypes varied in the number and size of glandular trichomes produced (Patzak et al. 2015). In *Solanum* spp., there was considerable variation in trichomes types, shape and density among different species (Watts and Kariyat 2021). Further investigations utilizing a range of different genotypes of cannabis with varying trichome densities, as reported here for two genotypes, and their resulting THC levels, are needed. While higher trichome densities may correlate with higher cannabinoid levels, other factors, such as the activity of THCA synthase and CBDA synthase in the heads, are likely to become limiting.

Maturation of the stalked-capitate trichomes (defined as the morphological and physiological stage of trichome development preceding senescence) is associated with several visual changes. First, cuticular thickening, a characteristic of advanced stages of capitate glandular head development, increases by nearly eightfold (Mahlberg and Kim 1991). Second, non-volatile or less volatile compounds are exuded onto the surface of the glandular head to form a sticky residue. The mechanism by which secretion occurs has not been determined, but it could be taking place by diffusion or through cracks and ruptures in the cuticle. The cuticular structure in plants allows passage of various types of molecules to occur, including polar compounds and certain nonpolar molecules (Fernandez et al. 2016). The migration of vesicles inside glandular heads towards the cuticle could be involved in secretion of resin externally (Supplementary Fig. 2). Extrusion of resin to form droplets on the surface of the glandular head was commonly observed, and this caused them to fuse or stick together. This was accompanied by a gradual shrinkage and eventual collapse of the glandular head. Third, trichome maturation is associated with a change in color of the contents within the head, from cloudy to amber, presumably as a result of oxidation of phenolic compounds within the trichome head. Mahlberg and Kim (2004) suggested that senescent (over-mature) trichome glands contained lower levels of THC due to secretion or volatilization of the cannabinoids. Fourth, UV autofluorescence is increased as trichome glandular heads mature (Livingstone et al. 2020), which was confirmed in the present study. Pure cannabinoids show fluorescence at a peak emission of 430 nm (Hazekamp et al. 2005), emitting a strong blue-shifted signal. After secretion of resin, the degree of glandular head autofluorescence diminishes. Fifth, detachment of the glandular head can occur upon maturation of the trichome, potentially leading to a decrease in total cannabinoid content. The development of a constricted zone at the top of the stalk caused many glandular heads to become dislodged from the stalks, especially during the post-harvest and drying phases. The evolutionary significance of glandular head detachment upon maturity is poorly understood.

The above maturation-associated factors could be important measures by which trichome maturity can be assessed and potentially used to predict the optimal time of harvest that best ensures trichome integrity and optimal THC level. The collapse of glandular heads following secretion of resin during senescence occurred at 7 to 8 weeks of inflorescence development (Fig. 11) and was visually correlated with the development of an amber-brown color within the glands. This color transition is considered by cannabis growers to result in over-mature inflorescences with reduced THC levels. Environmental stresses, such as excessive heat or drought stress during inflorescence development, can lead to premature trichome browning and senescence of glandular heads (authors, unpublished observations). This can occur as early as 5 weeks of the flowering period (Fig. 11), when trichomes are not fully mature. The scanning electron micrographs from this study illustrate for the first time the morphological changes that take place during glandular head secretion and senescence. Early and excessive resin secretion can cause a reduction in final THC levels measured in dried inflorescence samples.

Commercially dried inflorescence samples of genotype “Pink Kush” were examined to determine the effect of hand-trim compared to machine-trim on the frequency of intact glandular heads. These two post-harvest handling methods, in which inflorescences are either trimmed to remove inflorescence leaves manually or mechanically in a trimmer (Caplan et al. 2022), respectively, was observed to affect the proportion of trichomes with intact/detached glandular heads. The hand-trim method provided a greater number of intact glandular heads compared to machine-trim. Therefore, handling methods can potentially introduce variability in trichome structure among commercially dried cannabis products intended for recreational or medicinal use.

The effect of cannabis genotype on trichome density (numbers per mm^2^), proportion of stalked:unstalked trichomes, and the proportion of trichomes that were dehisced (no glandular head) or senescent (glandular heads visibly shriveled) was also examined in dried samples. Differences in these parameters were noticeable, depending on the genotype. The drying process caused trichome stalks to appear shriveled and twisted although the majority of the glandular heads were not shriveled or damaged. Furthermore, the drying process did not impact the integrity of the cuticle surrounding the glandular heads as it is conducted at moderate temperatures (21–24 °C). High-temperature drying (50–70 °C) of leaves of pepper-rosmarin was shown to disrupt trichomes and resulted in a loss of essential oils (Queiroz et al. 2018). In basil, different drying methods caused changes in trichome integrity and affected essential oil content (de Santana et al. 2014). Forced air drying in an oven disrupted the cuticle of the glandular head and caused a reduction in essential oil content as compared to air-drying at lower temperatures, such as that conducted for cannabis. In hop cones, drying and post-harvest processing was also reported to cause damage to the glandular trichomes as observed using scanning electron microscopy (Srečec et al. 2011a). There have been no previous studies to assess the impact of commercial drying conditions on the integrity of trichomes in *Cannabis*. The trichome density, ratio of sessile-capitate and stalked-capitate trichomes, as well as the proportion that had secreted resin or had become detached in dried samples was influenced by the cannabis genotype. Furthermore, the integrity of stalked-capitate trichomes at maturity was affected more by the post-harvest handling practice than the drying process itself and can potentially impact THC or CBD levels. While microscopic examination of dried cannabis flower samples for trichome head integrity is difficult to perform routinely, quality assurance criteria which includes assessing glandular head numbers and maturation may be important parameters by which to assess the quality of the cannabis product.

## Conclusion

The results from this study demonstrate that light and scanning electron microscopy can provide insightful information on cannabis trichome development and maturation. By obtaining a better understanding of the influence of genetic and environmental factors on trichome maturation, this could be used to predict the optimal time of inflorescence harvest, i.e., the time at which maximum potential THC levels could be attained. The use of a combination of visual markers, such as changes in glandular head color, increased autofluorescence, greater stalk lengths and head diameter, as described in this study, or as-yet-undetermined biochemical markers, can provide valuable insights to ensure product consistency. The asynchronous development and maturation of stalked-capitate trichomes could present a challenge in achieving a consistent predicted measure of maturation. Understanding the intricate interplay of genotype, environmental stress factors, and exogenous factors, such as hormones, and their effects on cannabis trichome development, and potentially cannabinoid levels, still requires further research which can potentially lead to significant commercial applications.

## Supplementary Information

**Additional file 1: Supplementary Figure 1.** Trichome development on leaves of genotype SQ of Cannabis sativa. a) A young leaf showing abundant formation of nonglandular trichomes, especially along the midveins. b) Close-up view of nonglandular trichomes (arrow). c) A mixture of nonglandular trichomes and sessile capitate trichomes on an older leaf. d) Close-up view showing nonglandular trichomes, sessile capitate trichomes (ss) and bulbous trichomes (bu) on an older leaf. Stalked-capitate trichomes were absent.

**Additional file 2: Supplementary Figure 2.** Secretion of resin from glandular heads. a, b) Highly magnified heads using macrophotography and phase contrast imaging showing distribution of vesicles inside the heads. A ring of secretory cells can be seen on the underside of the heads above the stalk. (Images are courtesy of 11 Zoom Gardens and are included with the acknowledgement of credit to the photographer Nick Cash). c, d) Scanning electron micrographs of the ring of secretory cells (c) and droplets of resin secreted on the outside of the cuticle (d). e) Microscopic pores (approx. 0.5 um in diameter) that presumably may allow resin to be secreted through the cuticle. The confirmation of the presence and function of these pores requires additional studies.

**Additional file 3: Supplementary Figure 3.** Analysis of images of trichomes using GIMP image processing and software and using pseudo-code for calculating translucency and red score for the image.

## Data Availability

Not applicable.
