# Supplementary figures and images for: Glandular trichome development, morphology, and maturation are influenced by plant age and genotype in high THC-containing cannabis (Cannabis sativa L.) inflorescences
Source: J Cannabis Res. 2023 Apr 4;5:12. doi: 10.1186/s42238-023-00178-9 (PMC10071647; doi:10.1186/s42238-023-00178-9)

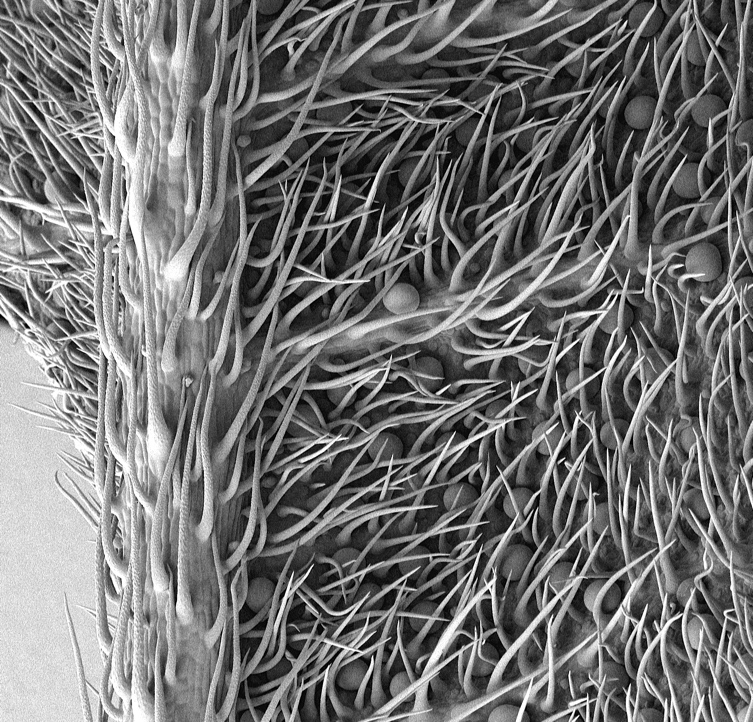

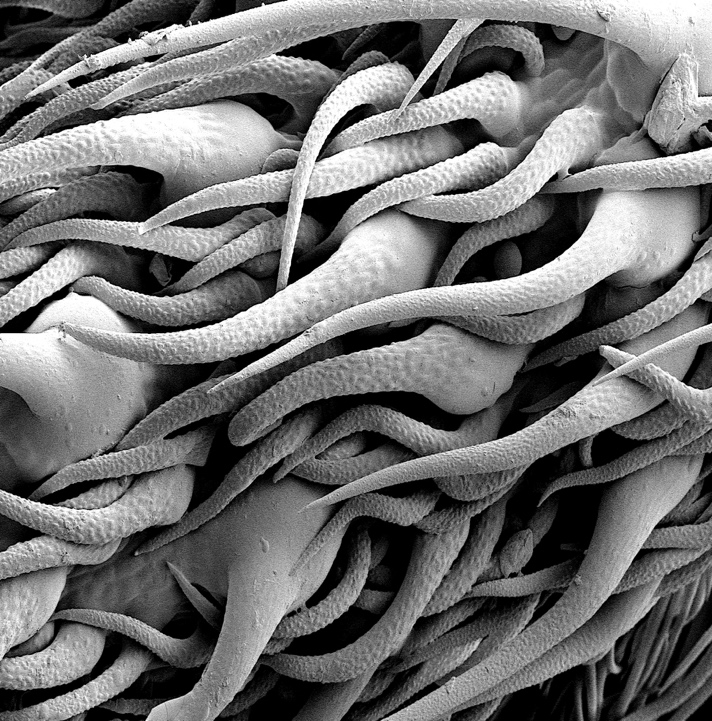


25 um

250 um

**b**

**a**


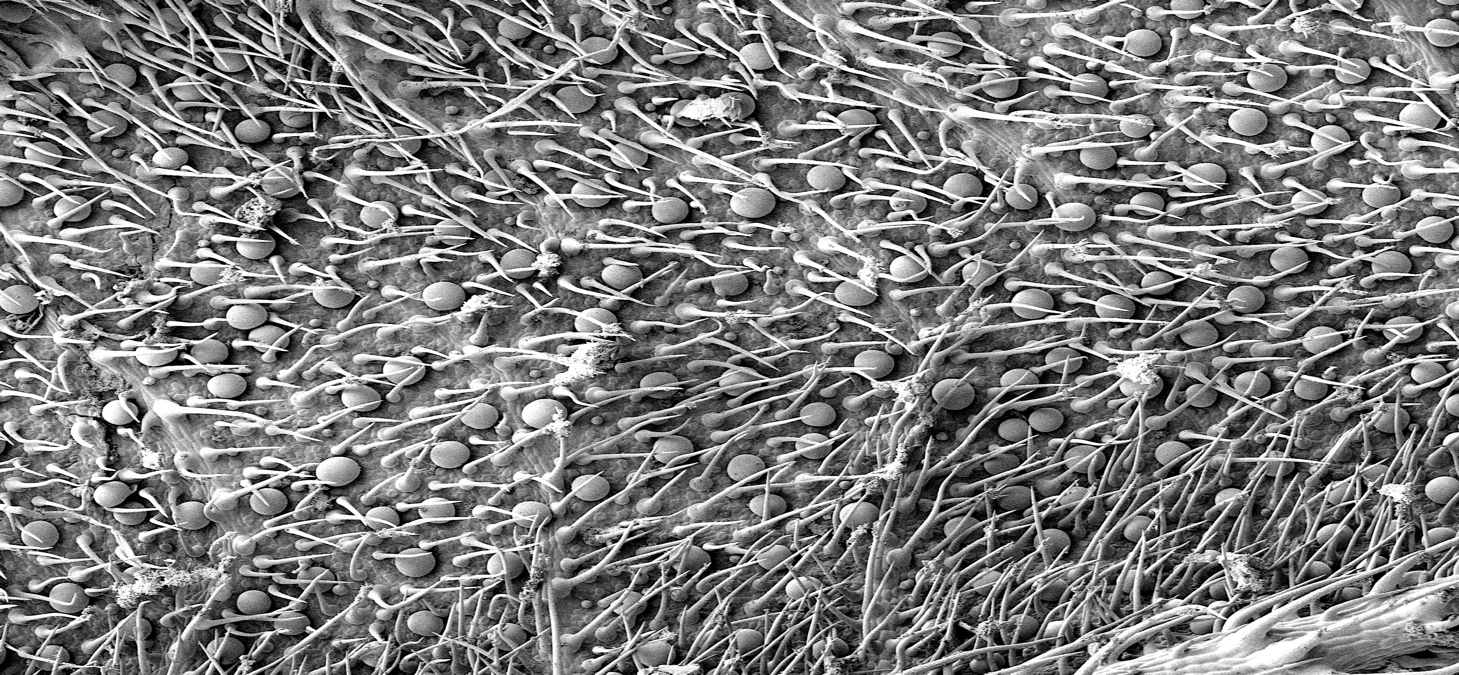


300 um

**c**


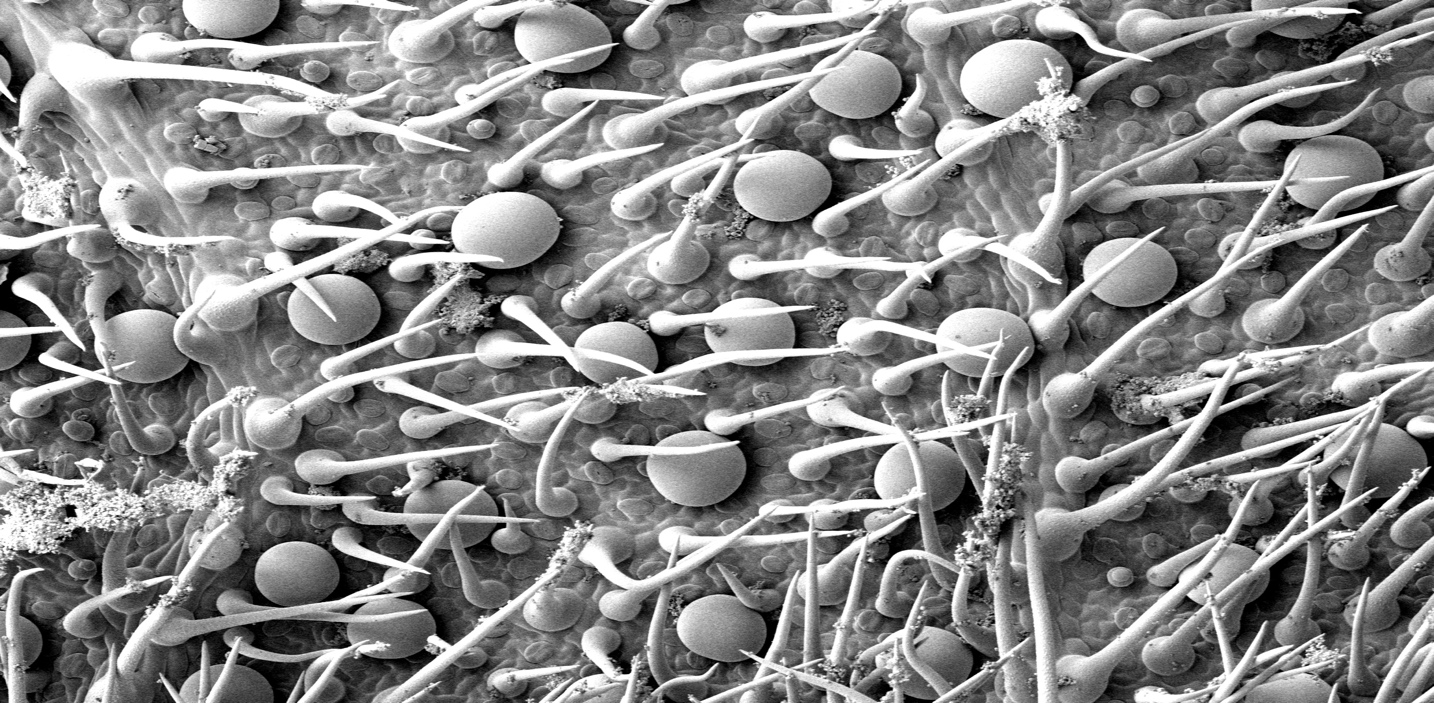


100 um

**d**

**ss**

**buuu**

Supplement: Supplementary file 1 — Additional file 1: Supplementary Figure 1. Trichome development on leaves of genotype SQ of Cannabis sativa. a) A young leaf showing abundant formation of nonglandular trichomes, especially along the midveins. b) Close-up view of nonglandular trichomes (arrow). c) A mixture of nonglandular trichomes and sessile capitate trichomes on an older leaf. d) Close-up view showing nonglandular trichomes, sessile capitate trichomes (ss) and bulbous trichomes (bu) on an older leaf. Stalked-capitate trichomes were absent. [file 42238_2023_178_MOESM1_ESM.docx]

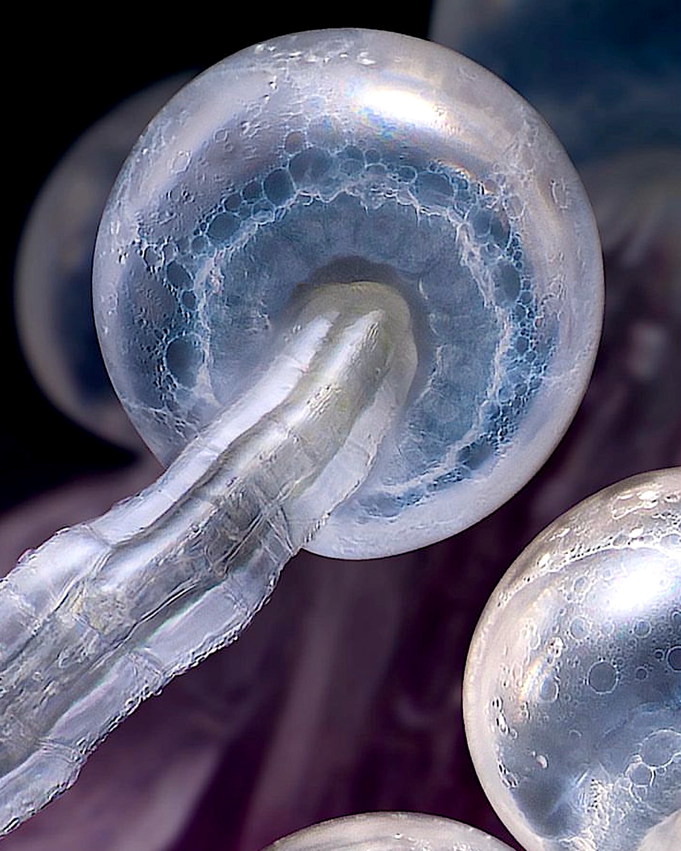

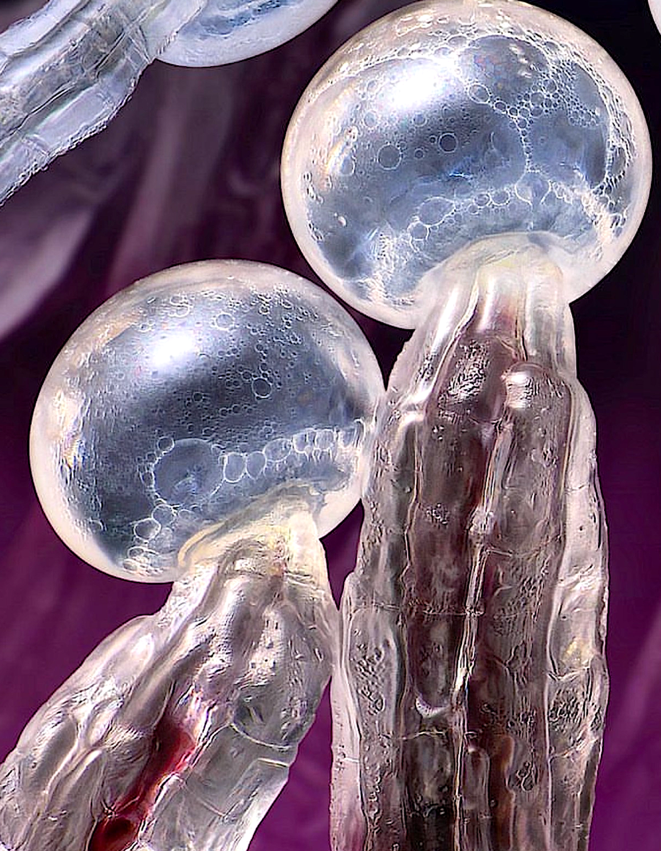


**b**

**a**

25 um

20 um


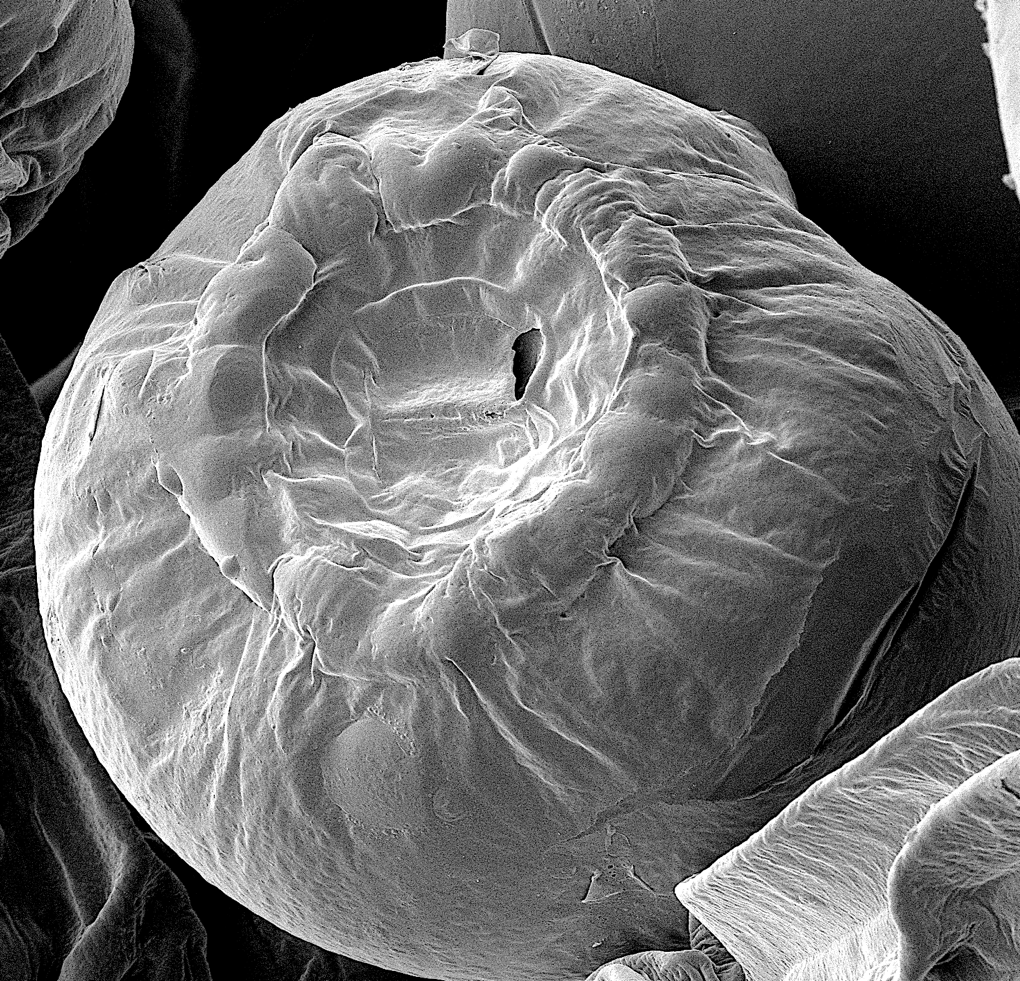

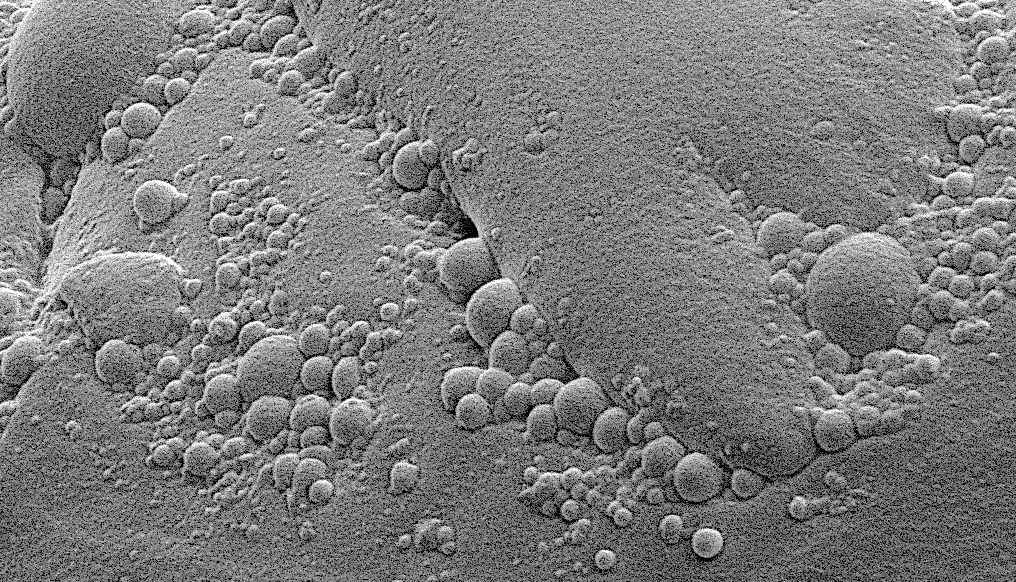


5 um

15 um

**c**

**d**


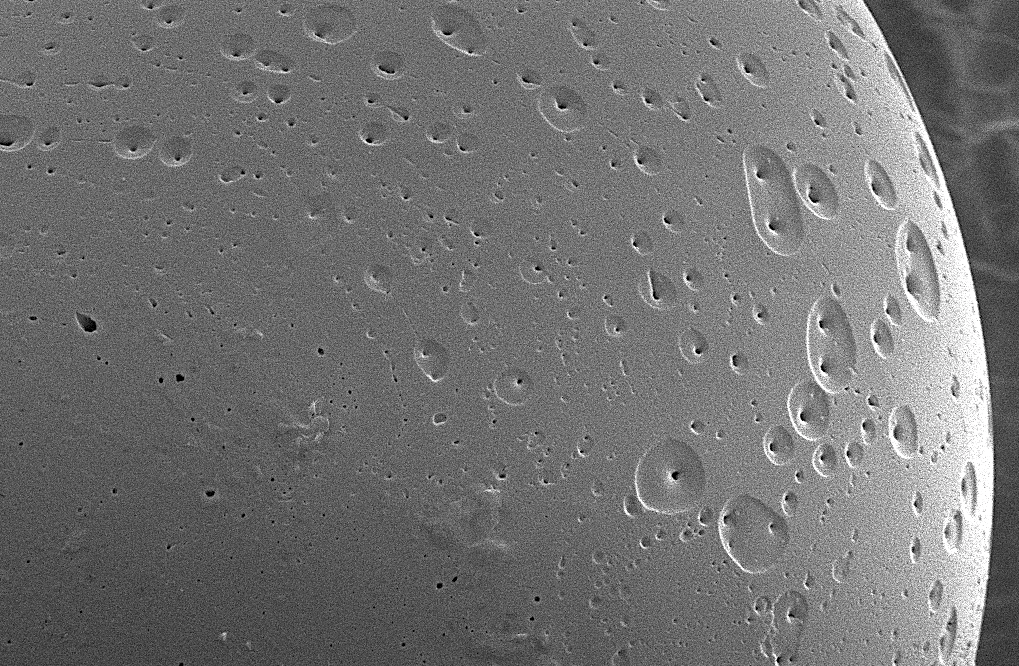


5 um

**e**

Supplement: Supplementary file 2 — Additional file 2: Supplementary Figure 2. Secretion of resin from glandular heads. a, b) Highly magnified heads using macrophotography and phase contrast imaging showing distribution of vesicles inside the heads. A ring of secretory cells can be seen on the underside of the heads above the stalk. (Images are courtesy of 11 Zoom Gardens and are included with the acknowledgement of credit to the photographer Nick Cash). c, d) Scanning electron micrographs of the ring of secretory cells (c) and droplets of resin secreted on the outside of the cuticle (d). e) Microscopic pores (approx. 0.5 um in diameter) that presumably may allow resin to be secreted through the cuticle. The confirmation of the presence and function of these pores requires additional studies. [file 42238_2023_178_MOESM2_ESM.docx]

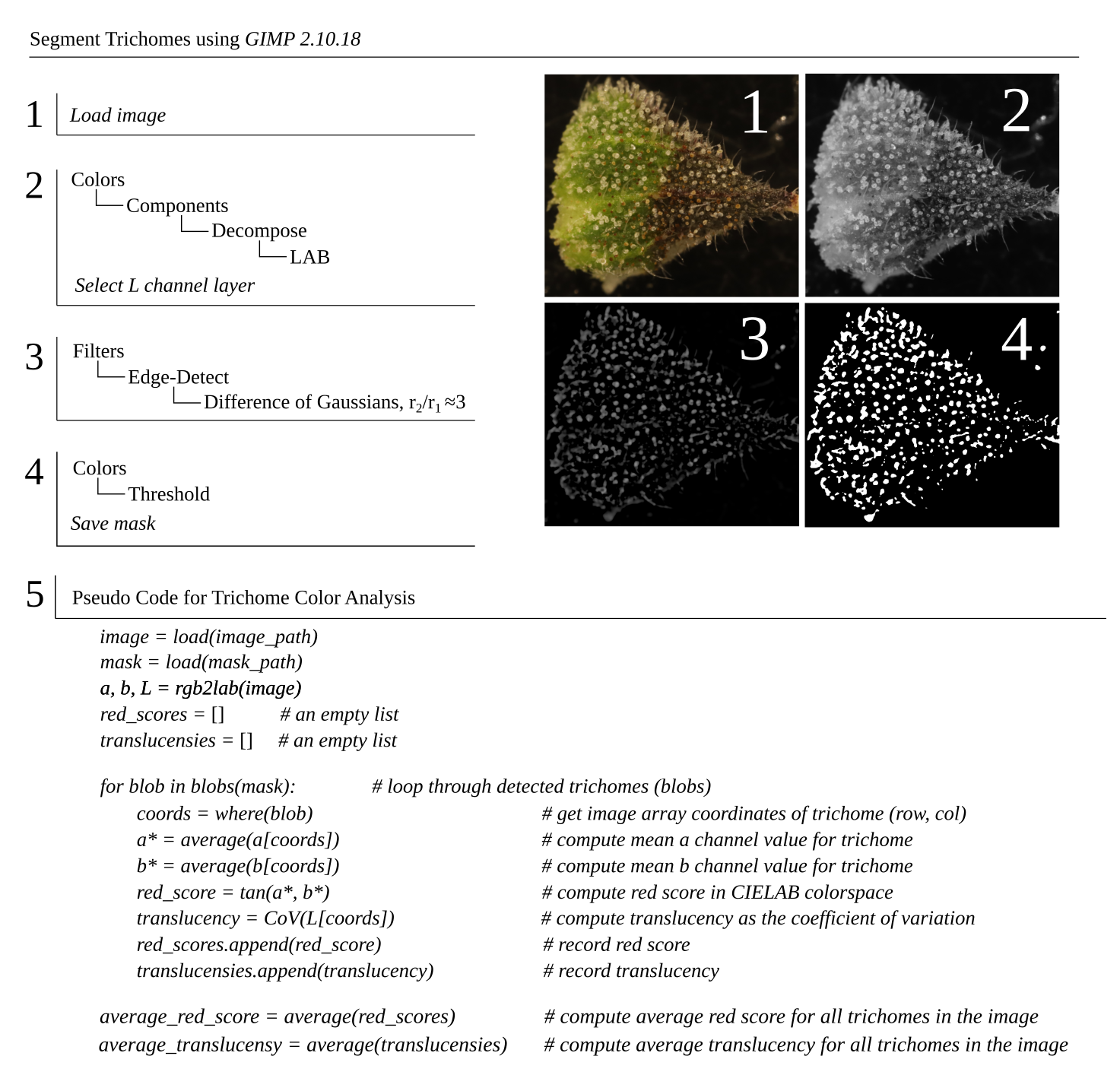


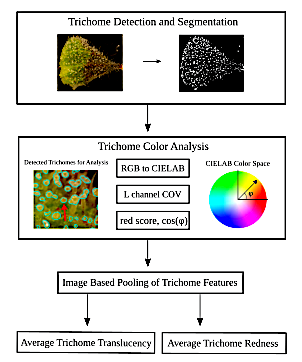

Supplement: Supplementary file 3 — Additional file 3: Supplementary Figure 3. Analysis of images of trichomes using GIMP image processing and software and using pseudo-code for calculating translucency and red score for the image. [file 42238_2023_178_MOESM3_ESM.docx]
